# iCOVID: interpretable deep learning framework for early recovery-time prediction of COVID-19 patients

**DOI:** 10.1038/s41746-021-00496-3

**Published:** 2021-08-16

**Authors:** Jun Wang, Chen Liu, Jingwen Li, Cheng Yuan, Lichi Zhang, Cheng Jin, Jianwei Xu, Yaqi Wang, Yaofeng Wen, Hongbing Lu, Biao Li, Chang Chen, Xiangdong Li, Dinggang Shen, Dahong Qian, Jian Wang

**Affiliations:** 1 School of Biomedical Engineering, Shanghai Jiao Tong University, Shanghai, China; 2 Department of Radiology, Southwest Hospital, Third Military Medical University (Army Medical University), Chongqing, China; 3 Department of Gastroenterology, Southwest Hospital, Third Military Medical University (Army Medical University), Chongqing, China; 4 Department of Radiation Oncology, Stanford University School of Medicine, Stanford, CA USA; 5 College of Media, Communication University of Zhejiang, Hangzhou, China; 6 College of Computer Science and Technology, Zhejiang University, Hangzhou, China; 7 Department of Nuclear Medicine, Ruijin Hospital, Shanghai, China; 8 Department of Thoracic Surgery, Shanghai Pulmonary Hospital, Tongji University School of Medicine, Shanghai, China; 9 Department of Radiology, General Hospital of Southern Theatre Command, PLA, Guangzhou, China; 10 Department of Radiology, Huoshenshan Hospital, Wuhan, China; 11 School of Biomedical Engineering, ShanghaiTech University, Shanghai, China; 12 Department of Research and Development, Shanghai United Imaging Intelligence, Co., Ltd, Shanghai, China

**Keywords:** Viral infection, Machine learning

## Abstract

Most prior studies focused on developing models for the severity or mortality prediction of COVID-19 patients. However, effective models for recovery-time prediction are still lacking. Here, we present a deep learning solution named iCOVID that can successfully predict the recovery-time of COVID-19 patients based on predefined treatment schemes and heterogeneous multimodal patient information collected within 48 hours after admission. Meanwhile, an interpretable mechanism termed FSR is integrated into iCOVID to reveal the features greatly affecting the prediction of each patient. Data from a total of 3008 patients were collected from three hospitals in Wuhan, China, for large-scale verification. The experiments demonstrate that iCOVID can achieve a time-dependent concordance index of 74.9% (95% CI: 73.6-76.3%) and an average day error of 4.4 days (95% CI: 4.2-4.6 days). Our study reveals that treatment schemes, age, symptoms, comorbidities, and biomarkers are highly related to recovery-time predictions.

## Introduction

Since the outbreak of coronavirus disease 2019 (COVID-19), artificial intelligence (AI) has played an essential role in the global fight against the pandemic, including (1) contactless telehealth systems for remote diagnosis to protect doctors and patients from the high risk of viral exposure^1^ and (2) computer-aided diagnosis of the infection based on X-ray or computed tomography (CT) images to reduce the workload of healthcare workers^2–9^. In clinical practice, it is routine for COVID-19 patients to undergo various laboratory examinations, such as blood tests, liver function tests, and CT scans. Meanwhile, patients may suffer from different symptoms^10,11^ and comorbidities^12^, producing large quantities of heterogeneous multimodal clinical data. Such heterogeneity represents a substantial challenge for clinicians aiming to manually analyze the complicated clinical information and provide an appropriate treatment scheme for patients. Consequently, there is a great need for automatic data analysis methods to aid clinical treatment planning for COVID-19, which has also received widespread attention over the past year^13–17^. Some studies have demonstrated that biomarkers, symptoms, comorbidities, and even CT images can be applied for various prognostic prediction tasks, including the prediction of mortality risk^18,19^, progression to a severe or critical state^17,20–22^, and intensive care unit admission^23,24^.

The modeling methods used in the above-mentioned studies can be roughly classified into the following two categories: (1) pure nonlinear methods^21,22^ and (2) linear and nonlinear hybrid methods^17,19,20^. The former directly construct deep learning models using heterogeneous multimodal data for specific tasks. For example, Ning et al.^22^ fused image features extracted by a deep convolutional neural network (DCNN) with other clinical features for severity-level prediction of patients. Deep learning methods can build a nonlinear relationship between the model inputs and the corresponding outputs, which can achieve promising performance. However, deep models are black boxes lacking the interpretability of the prediction results^25^. Generally, clinicians are eager to know the clinical factors that are highly related to the prediction result rather than simply the prediction result. In contrast, hybrid methods are more practical. These methods usually first use linear analysis methods (e.g., multivariable regression or LASSO regression) to select statistically linear-significant clinical features and then train machine learning or deep learning models via the preselected features. However, these statistical analysis methods still cannot provide individual interpretability of the model prediction of each patient.

More importantly, the disease status of COVID-19 patients changes over time, i.e., a dynamic process of mutual influence between treatments and patient covariates (i.e., symptoms, comorbidities, and biomarkers)^26^. However, treatment information was not considered in the model developed in most prior studies, and the models were only implemented as classification tasks^27,28^, e.g., severity-level classification. A more practical model should focus on directly predicting the recovery time of patients based on treatment information rather than only classifying patients’ severity levels. However, it is challenging to construct models for this purpose mainly due to the following complicated characteristics of clinical data sets: (1) data sets contain a large proportion of patients with unknown outcomes who were transferred to other hospitals and thus lost to follow-up (so-called censored data in the survival analysis field). Thus, how to reasonably utilize these data when constructing models for recovery-time prediction remains problematic; and (2) there are individual differences in recovery times among similar patients. For example, two similar patients might have different recovery times even if they were treated with identical treatment schemes. It might be difficult to converge a model at the training stage owing to this time-variant issue. To avoid the above-mentioned issues, Cox’s proportional hazard (CPH) model, which is the most commonly used method in the survival analysis field^29^, assumes a time-invariant linear combination of patients’ clinical features to simplify the model construction at the cost of poor performance.

**Fig. 1:**
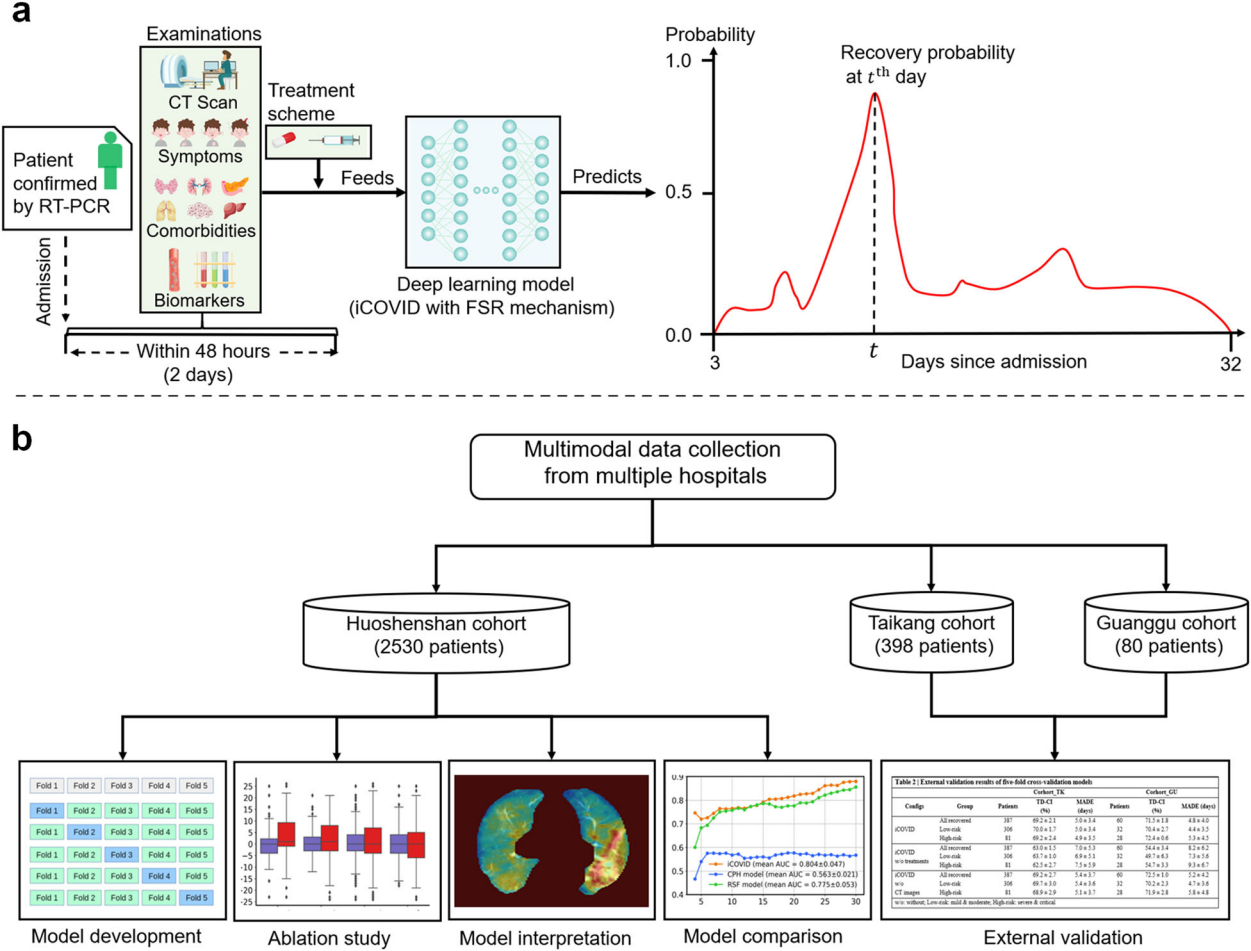
Prognostic model development and study design for recovery-time prediction. **a** Multimodal clinical data of a patient (i.e., CT images, symptoms, comorbidities, and biomarkers acquired within 48 hours after admission) are fed into a deep learning model to predict recovery probabilities in a time range. **b** Development and validation of the deep learning model based on multicenter cohorts.

In this study, we present an end-to-end deep learning framework termed iCOVID that considers treatment information for the early prediction of COVID-19 recovery time (Fig. 1a). iCOVID can fully use heterogeneous multimodal data (i.e., CT images, biomarkers, symptoms, comorbidities, and treatment information) from patients with different outcomes to learn the time-variant nonlinear relationship between the data and predictions. Furthermore, a feature significance ranking (FSR) mechanism is proposed to learn the nonlinear regression coefficients reflecting the significance of each feature to the prediction outputs. Extensive experiments based on multicenter data are performed to demonstrate the effectiveness of the proposed method (Fig. 1b).

The main contributions of this study can be summarized as follows: (1) we develop a deep learning method (i.e., the iCOVID) for recovery-time prediction of COVID-19 patients based on a large quantity of multimodal clinical data. Particularly, treatment information is considered an important factor in our work. (2) The proposed iCOVID is a time-dependent regression model, rather than a classification model, that can predict a “recovery probability distribution” within a time range since admission (see Fig. 1a). (3) An interpretable mechanism (i.e., the FSR) is designed to learn the significance of clinical features in an end-to-end manner, thereby avoiding the preselection of features.

## Results

### Data acquisition and preparation

**Fig. 2:**
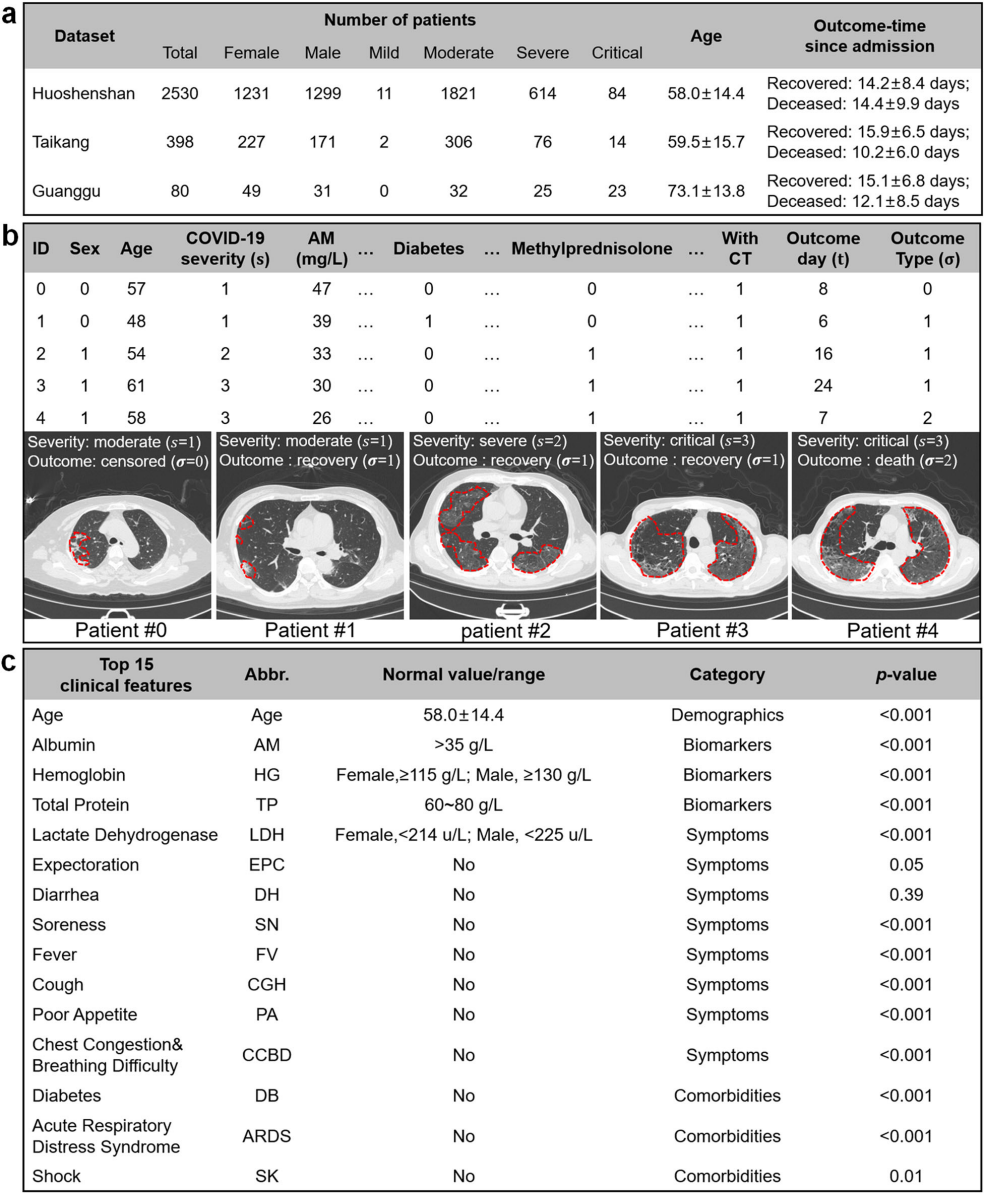
Data set information. **a** Patient information of the three cohorts. **b** Five samples of the patients’ tuple information. **c** The top 15 clinical features identified via the proposed FSR mechanism that are significant to the recovery-time prediction of COVID-19 patients. *p* values calculated via a Pearson correlation analysis demonstrate that these features are indeed highly related to the recovery time.

To develop and evaluate iCOVID, we built a relatively large-scale data set containing retrospective data collected from a total of 2530 COVID-19 patients from Huoshenshan Hospital in Wuhan, China. From each patient, we collected the following information: (1) used treatment schemes, (2) primitive CT scans, (3) clinical features, (4) severity-level, (5) patient outcome (recovered, decreased, or censored), and (6) outcome occurring days since admission. Each treatment scheme consisted of 19 types of drugs or treatment tools, while the clinical features included two demographics (age and gender), 10 types of symptoms, 7 types of comorbidities, and 27 types of biomarkers (Supplementary Tables 1–2). In this study, all patients were randomly divided into subsets for fivefold cross-validation (Supplementary Fig. 1d). To test the generalization of iCOVID, we also built two additional cohorts as external validation sets with data collected from two hospitals in the epicenter of Wuhan (Taikang Tongji Wuhan Hospital, and Hubei Maternity and Child Healthcare Guanggu Hospital). The patient statistics are summarized in Fig. 2a and Supplementary Fig. 1a–c. More details of the data acquisition are provided in the Methods section.

Formally, each patient can be defined as a tuple $\left( {\overrightarrow {\boldsymbol{\tau}} _n,{{{\boldsymbol{I}}}}_n,\overrightarrow {\boldsymbol{x}} _n,s_n,\sigma _n,t_n} \right)$ representing the above-mentioned six categories of information. Here, $\overrightarrow {\boldsymbol{\tau}} _n$ is a 19-dimensional vector of treatment schemes, with each element represented by a binary value of 1 or 0, indicating whether a specific treatment or drug was used for the patient. ***I***_*n*_ is an image matrix of the CT scan. $\overrightarrow {\boldsymbol{x}} _n$ refers to a vector consisting of the 46 clinical feature values. *s*_*n*_ is an indicator of the severity-level as follows: mild|*s*_*n*_ = 0, moderate|*s*_*n*_ = 1, severe|*s*_*n*_ = 2, and critical|*s*_*n*_ = 3. *σ*_*n*_ is an indicator of the outcome type as follows: censored data |*σ*_*n*_ = 0, recovery|*σ*_*n*_ = 1, and death|*σ*_*n*_ = 2. *t*_*n*_ is the day on which the outcome occurred. Figure 2b shows some examples of the tuple information. Figure 2c lists the top 15 clinical features identified via the FSR mechanism, which are highly related to the recovery-time prediction of COVID-19 patients.

### Network architecture for recovery-time prediction

**Fig. 3:**
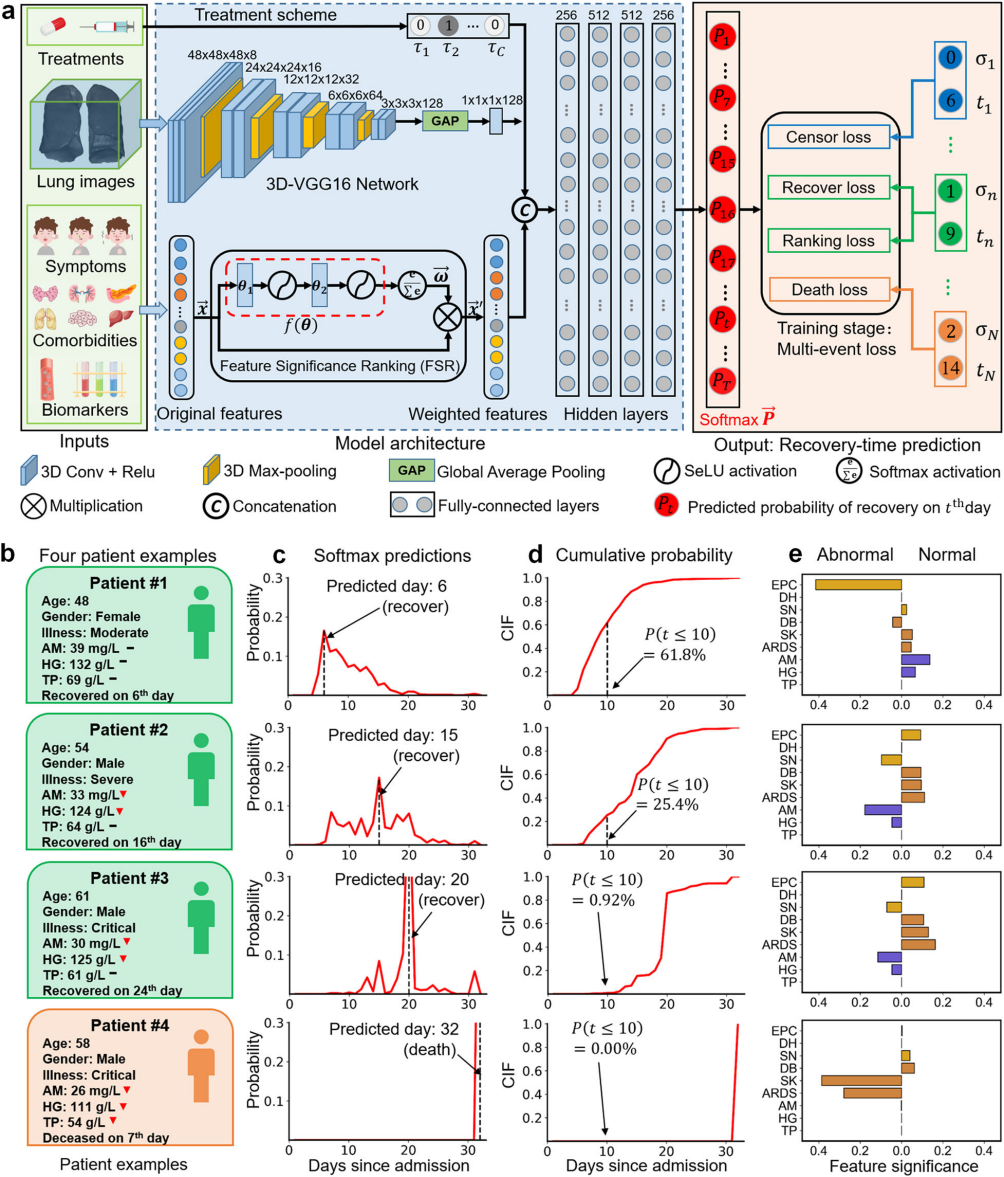
Framework architecture and predictions of four patient examples. **a** The treatment schemes, lung CT images, and clinical features of the patients are fed to the framework for recovery-time prediction. The FSR subnetwork is designed to learn the significance of each clinical feature that contributes to the predictions. A multi-event loss is designed to train the model using data from patients with different outcomes. **b** The information of the patients. **c** The softmax outputs (i.e., ${\overrightarrow {\boldsymbol{P}}}$ in **a**) of the four patients. The days a patient needs to recover can be estimated by the day with the maximum probability. **d** The cumulative probability of the softmax outputs, which can be used to assess the risk of patients. **e** The top nine clinical features that are significant for the decision made by the model (*EPC* expectoration, *DH* diarrhea, *SN* soreness, *DB* diabetes, *SK* shock, *ARDS* acute respiratory distress syndrome, *AM* albumin, *HG* hemoglobin, *TP* total protein).

Figure 3a illustrates the main architecture of iCOVID, which incorporates treatment schemes, lung CT images, and clinical features as inputs. Convolutional features are extracted from the lung images using the VGG-16 network^30^, which are then combined with clinical features and treatment schemes using fully connected layers for recovery-time prediction. The output component is a softmax layer with *T* neurons estimating a probability distribution ${\overrightarrow {P}} = \left[ {P_1, \ldots ,P_t, \ldots ,P_T} \right]$ within a predefined day range {1, 2, …, *T*} for each patient. In this expression, each element $P_t \in [0,1]$ indicates the possibility of recovery on the *t*^th^ day after admission. Considering that the number of patients who required >30 days to recover was generally low (see Supplementary Fig. 1c), we assumed that the recovery day of patients who recovered after 30 days was 31 and that of patients who died was 32. Hence, the maximum day *T* was set to a value of 32.

To address the “black box” issue of the deep model, the FSR mechanism is incorporated in the framework as a subnetwork to estimate the significance of each clinical feature for the final predictions. Specifically, the FSR can automatically produce a weighting vector (denoted by ${\overrightarrow {\boldsymbol{\omega}}} = \left[ {\omega _1,\omega _2, \ldots ,\omega _K} \right],K = 46$) for each input clinical feature vector (i.e., ${\overrightarrow {\boldsymbol{x}}} = \left[ {x_1,x_2, \ldots ,x_K} \right],K = 46$**)**, where each element in the weighting vector represents the significance of the corresponding clinical feature. This mechanism allows us to determine the most significant clinical features for the prediction of each patient. The FSR can be trained with the whole framework end-to-end using a multi-event loss function that comprises four losses, i.e., the censor, recover, death, and ranking losses in Fig. 3a. The former three losses are designed to address censored, recovered, and decreased patients, whereas the ranking loss^31^ is introduced to address the time-variant issue among recovered patients. The relevant details are further discussed in the Methods section.

In clinical applications, the probability distribution (i.e., the softmax output ${\overrightarrow {\boldsymbol{P}}}$) produced by iCOVID can aid in visually assessing the risk of patients. Figure 3c plots the probability distribution of four patients as follows: patients #1–#3 recovered on the 6^th^, 15^th^, and 20^th^ day, and patient #4 died on the 13^th^ day after admission (see Fig. 3b; the black horizontal lines and the red triangles indicate normal and abnormal biomarkers, respectively). The predicted recovery day of each patient can be estimated by the specific day with the maximum probability, i.e., ${{{\mathrm{argmax}}}}( {\overrightarrow {\boldsymbol{P}} })$ (see the peak highlighted by the vertical dashed lines in Fig. 3c). In addition, calculating the cumulative incidence function (CIF measures the possibility of a patient recovering within a specific time range, see Eq. 4 in Methods) can assess patient risk more reliably. For example, patient #2 can be considered to have a higher risk than patient #1, as the CIF $P\left( {t \le 10} \right) = 25.4{{{\mathrm{\% }}}}$ of patient #2 is much smaller than $P\left( {t \le 10} \right) = 61.8{{{\mathrm{\% }}}}$ of patient #1 (see Fig. 3d). Figure 3e demonstrates the significance of nine clinical features obtained via the FSR (three for symptoms, comorbidities, and biomarkers each), revealing the important features corresponding to each patient’s prediction (the box length indicates the significance of the corresponding feature). It can be observed that the biomarker albumin (AM) and hemoglobin (HG) are important for the prediction of recovered patients #1–#3, whereas the comorbidity shock (SK) and acute respiratory distress syndrome (ARDS) play a more significant role in the identification of deceased patient #4.

### Impact of treatment schemes and CT images on recovery-time prediction

We evaluated the model performance quantitatively by calculating the time-dependent concordance index (TD-CI)^32^, which is a variant of the ordinary concordance index (CI) that is widely used as a discriminative index for prognostic estimation. In contrast to the CI, the TD-CI considers time and thus can reflect the potential change in outcome over time (see Eq. 8 in Methods). A larger value of TD-CI indicates the superior performance of the model. Furthermore, we assume that the predicted recovery day of each patient is the day with the maximum probability in the day range (see Fig. 3c). Then, to further validate the performance, we calculated the mean absolute day error (MADE) between the predicted and real recovery day of all recovered patients. Intuitively, the smaller the MADE value, the better the prediction of the model.

To validate the impact of treatment schemes and CT images on the prediction, we designed ablation experiments of iCOVID without using any treatment scheme or any CT images (clinical features, i.e., demographics, symptoms, comorbidities, and biomarkers were used as baseline information in all models, see Methods). The statistical results tabulated in Table 1 reveal that iCOVID can achieve promising performance with a TD-CI value as high as 74.9% (95% CI: 73.6%–76.3%) and a MADE value as low as 4.4 days (95% CI: 4.2–4.6 days) for all 1969 recovered patients. However, when the treatment scheme is ignored, the performance considerably worsens (TD-CI = 69.1% and MADE = 6.0 days). This phenomenon demonstrates that the treatment scheme is indeed an important factor in estimating how long a patient requires to recover. In addition, the results demonstrate that iCOVID also achieves inferior performance in both the TD-CI and MADE metrics when CT image information is ignored. However, the absolute difference is only 0.3% for TD-CI and 0.3 days for MADE, indicating that CT images are not as significant as treatment schemes for recovery-time prediction. Alternative DCNNs, such as ResNet-34^33^, MobileNet-v3^34^, InceptionNet-v4^35^, and EfficientNet-b3^36^, were also adopted as CNN feature extractors. The experimental results show that the choice of CNN model only has a trivial influence on the overall performance (see Supplementary Table 4).

**Table 1:** Fivefold cross-validation results: impact of treatments and CT images.

		iCOVID		iCOVID w/o treatments		iCOVID w/o CT images	
Subsets	Patients	TD-CI (%)	MADE (days)	TD-CI (%)	MADE (days)	TD-CI (%)	MADE (days)
Cohort_1	394	76.8 (74.0–79.4)	**4.2** (3.9–4.4)	70.8 (66.9–74.5)	5.8 (5.3–6.3)	**77.2** (74.4–80.0)	4.5 (4.1–4.9)
Cohort_2	394	**78.1** (75.5–80.5)	**4.1** (3.7–4.3)	69.8 (66.6–74.1)	6.1 (5.6–6.4)	77.0 (74.4–79.6)	4.3 (3.9–4.7)
Cohort_3	394	**75.3** (72.3–79.0)	**4.8** (4.5–5.2)	69.2 (66.0–72.8)	5.9 (5.5–6.4)	74.4 (71.5–77.3)	5.0 (4.7–5.3)
Cohort_4	394	76.0 (73.2–79.1)	**4.3** (4.0–4.6)	70.4 (66.3–74.2)	6.2 (5.7–6.6)	**76.8** (73.9–79.4)	**4.3** (4.0–4.7)
Cohort_5	393	**73.5** (70.3–76.6)	**4.7** (4.3–5.0)	67.4 (63.3–73.3)	5.8 (5.4–6.1)	72.0 (67.9–74.9)	5.2 (4.8–5.6)
Overall	1969	**74.9** (73.6–76.3)	**4.4** (4.2–4.6)	69.1 (67.7–70.5)	6.0 (5.7–6.2)	74.6 (72.8–76.1)	4.7 (4.5–4.8)

*w/o* without; the best performance in each row is shown in bold; (-) is the 95% confidence interval.

**Fig. 4:**
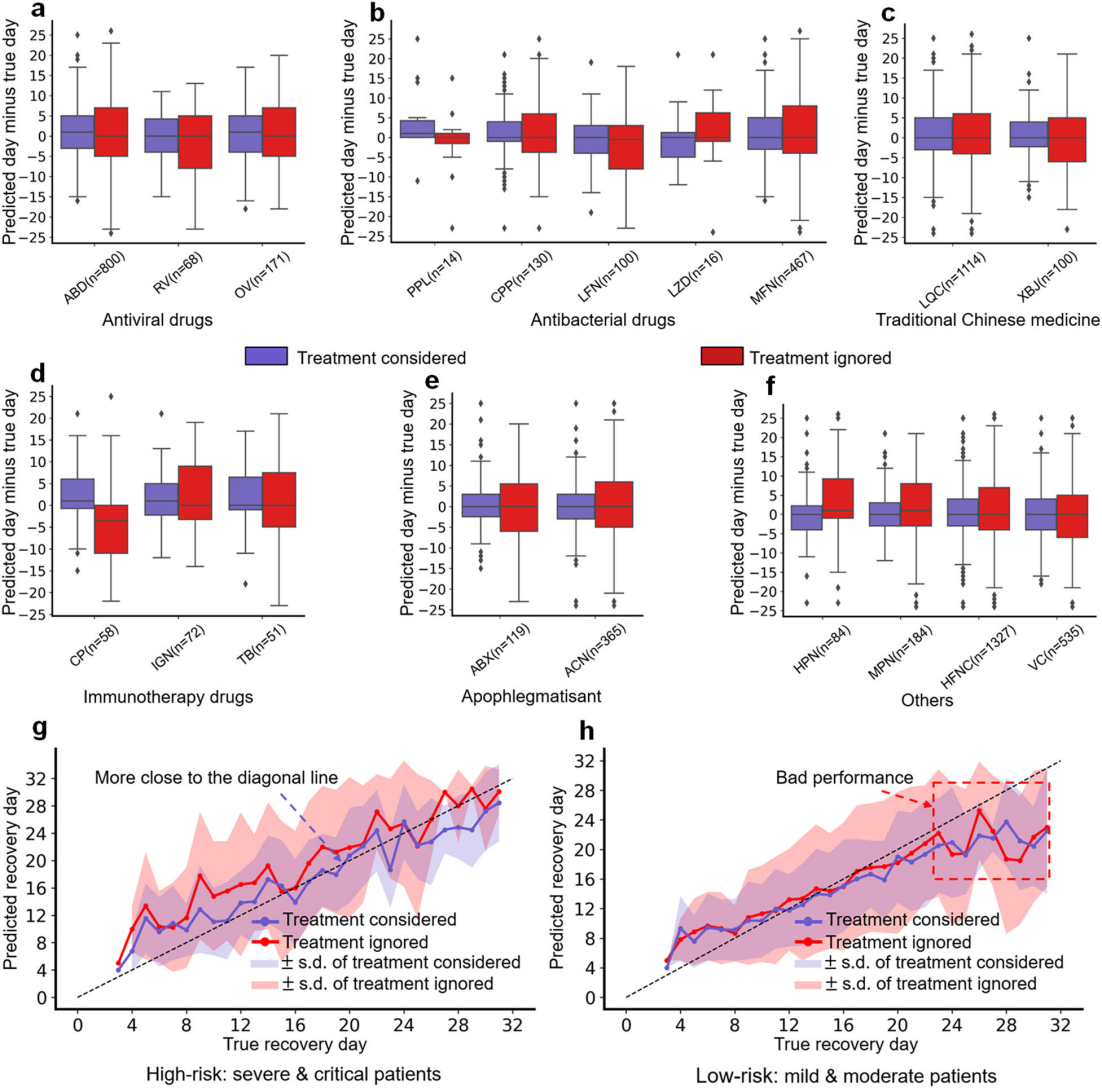
Distribution and statistics of the day error between the average predicted and true recovery days. **a**–**f** Plots of the day error statistics of patients corresponding to each treatment/drug group: antiviral drugs (*ABD* arbidol, *RV* ribavirin, and OV oseltamivir), antibacterial drugs (*PPL* piperacillin, *CPP* cephalosporins, *LFN* levofloxacin, *LZD* linezolid, and *MFN* moxifloxacin), traditional Chinese medicine (*LQC* Lianhua Qingwen Capsule and *XBJ* Xuebijing), immunotherapy drugs (*CP* convalescent plasma, *IGN* immunoglobulin, and *TB* tocilizumab), apophlegmatisant (*ABX* ambroxol and *ACN* acetylcysteine), and others (*HPN* heparin, *MPN* Methylprednisolone, *HFNC* high-flow nasal cannula oxygen; and *VC* Vitamin C). The centerline and the bounds of each box correspond to the median value and the interquartile range, respectively, and the whiskers mark the range of the non-outlier data. **g** iCOVID can estimate the recovery days of high-risk patients more accurately by considering treatment schemes. **h** Main prediction error for low-risk patients is derived from the patients who recovered after 24 days.

Figure 4a–f plot the day error statistics of patients corresponding to different treatment/drug groups: antiviral drugs (*ABD*: arbidol; *RV*: ribavirin; and *OV*: oseltamivir), antibacterial drugs (*PPL*: piperacillin; *CPP*: cephalosporins; *LFN*: levofloxacin; *LZD*: linezolid; and *MFN*: moxifloxacin), traditional Chinese medicine (*LQC*: Lianhua Qingwen capsule and *XBJ*: Xuebijing), immunotherapy drugs (*CP*: convalescent plasma; *IGN*: immunoglobulin; and *TB*: tocilizumab), apophlegmatisant (*ABX*: ambroxol and *ACN*: acetylcysteine), and others (*HPN*: heparin; *MPN*: methylprednisolone; *HFNC*: high-flow nasal cannula oxygen; and *VC*: vitamin C). It can be observed that the median values of most boxes are very close to zero, regardless of whether the treatment schemes are considered (dark-blue boxes) or not (red boxes). This phenomenon confirms the effectiveness of iCOVID in the recovery-time prediction of COVID-19 patients. Although the median values in most dark-blue boxes are similar to their counterparts, the main difference is that almost all dark-blue boxes have much smaller interquartile ranges than the red boxes. This finding proves that iCOVID can indeed achieve more stable predictions by considering treatment schemes. It is recognized that different patients might be treated using various treatment schemes. For example, critically ill patients normally received more treatments (Supplementary Fig. 2a). The number of treatment schemes might be an implication for the model predictions. However, our experimental results demonstrate that the treatment rather than the number of treatments is more significant on the prediction (Supplementary Fig. 2b).

We also analyzed the distribution of the average day error between the predicted and real recovery days in the following different patient groups: high-risk (severe and critical, Fig. 4g) and low-risk (mild and moderate, Fig. 4h) patients. The performance of iCOVID considering treatment schemes was much better than that without considering treatment schemes, especially for high-risk patients (Fig. 4g). In addition, both prediction performances were reduced for low-risk patients who recovered 24 days after admission (Fig. 4h). We attribute this issue to the constructed data set in which the number of collected patients who recovered after 24 days is very limited (Supplementary Fig. 1c), increasing the difficulty in estimating their recovery days.

### Model interpretation and significant clinical features for the prediction

**Fig. 5:**
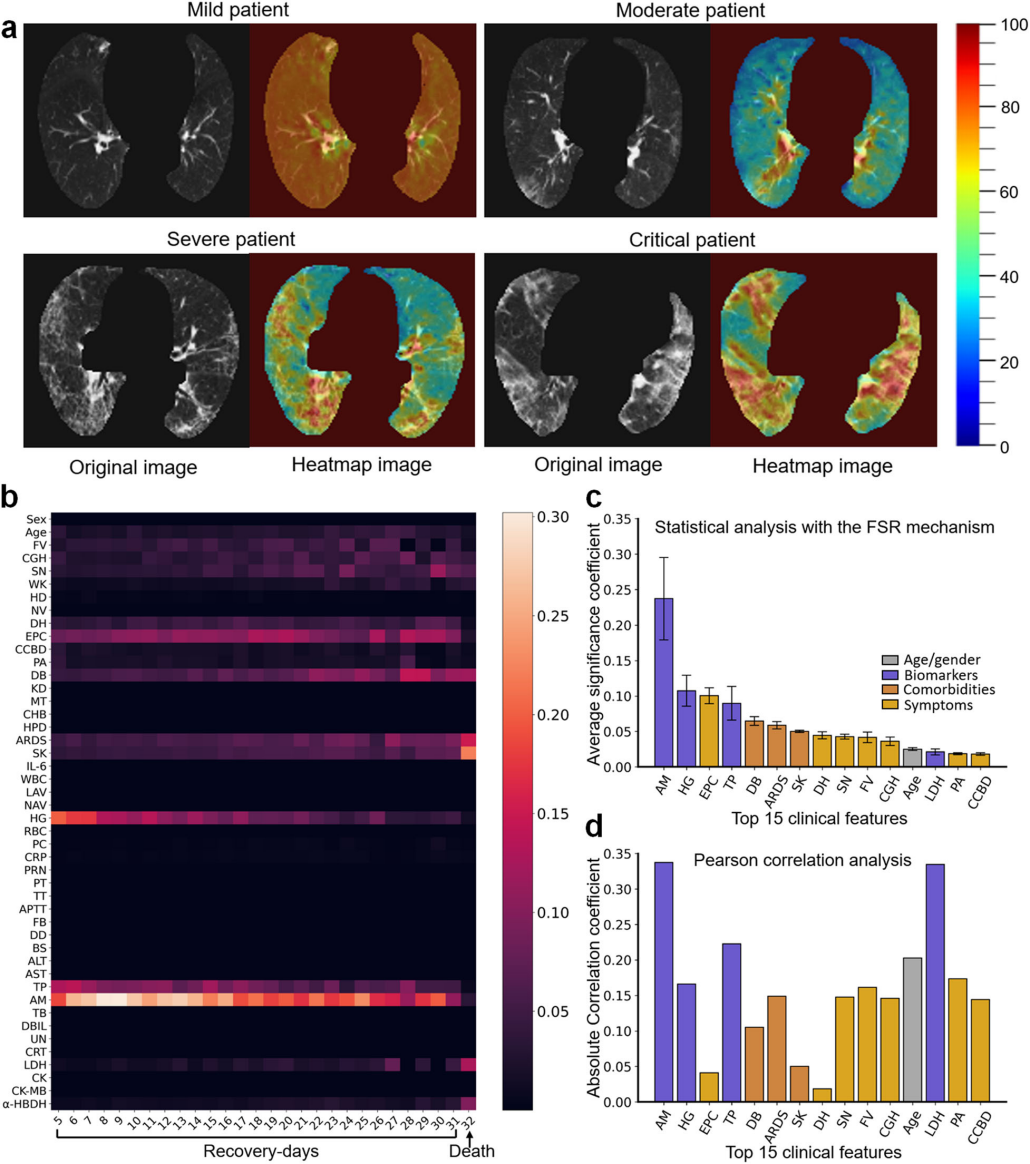
Network visualization and statistics of the feature significance for the model prediction. **a** Feature map visualization of iCOVID corresponding to four representative patient examples (color masks the significant regions for the prediction, with the spectrum from blue to red associated with low-to-high significance). **b** Heatmap of the average significance of each feature and the recovery days, revealing that the biomarkers *AM*, *TP*, and *HG* are significant for the prediction of recovered patients, whereas the comorbidities *SK*, *ARDS*, and *DB* are more significant for the prediction of deceased patients (color indicates the significance of each feature for the prediction, with the spectrum from dark purple to yellowish-white associated with low-to-high significance). **c** The average significance of the top 15 clinical features, i.e., *AM* albumin, *HG* hemoglobin, *EPC* expectoration, *TP* total protein, *DB* diabetes, *ARDS* acute respiratory distress syndrome, *SK* shock, *DH* diarrhea, *SN* soreness, *FV* fever, *CGH* cough, *LDH* lactate dehydrogenase, *PA* poor appetite, *CCBD* chest congestion/breathing difficulty. **d** Pearson correlation analysis demonstrates that the above-mentioned features are indeed highly related to the recovery time of COVID-19 patients (*p* value < 0.001, except for *EPC*, *DH*, and *SK*).

To understand the regions of the image and the types of clinical features that are highly related to the recovery-time prediction, we visualized convolutional feature maps using the Grad-CAM^37^ technique and calculated the average significance of each clinical feature based on the FSR mechanism. Figure 5a shows feature maps of four representative patients and demonstrates that the proposed model mainly focused on the lesion regions of CT images to make decisions regarding moderate, severe, or critical patients. Among mild patients, almost no lesion can be observed on CT images, and the proposed model mainly relied on the whole lung region to make predictions.

**Fig. 6:**
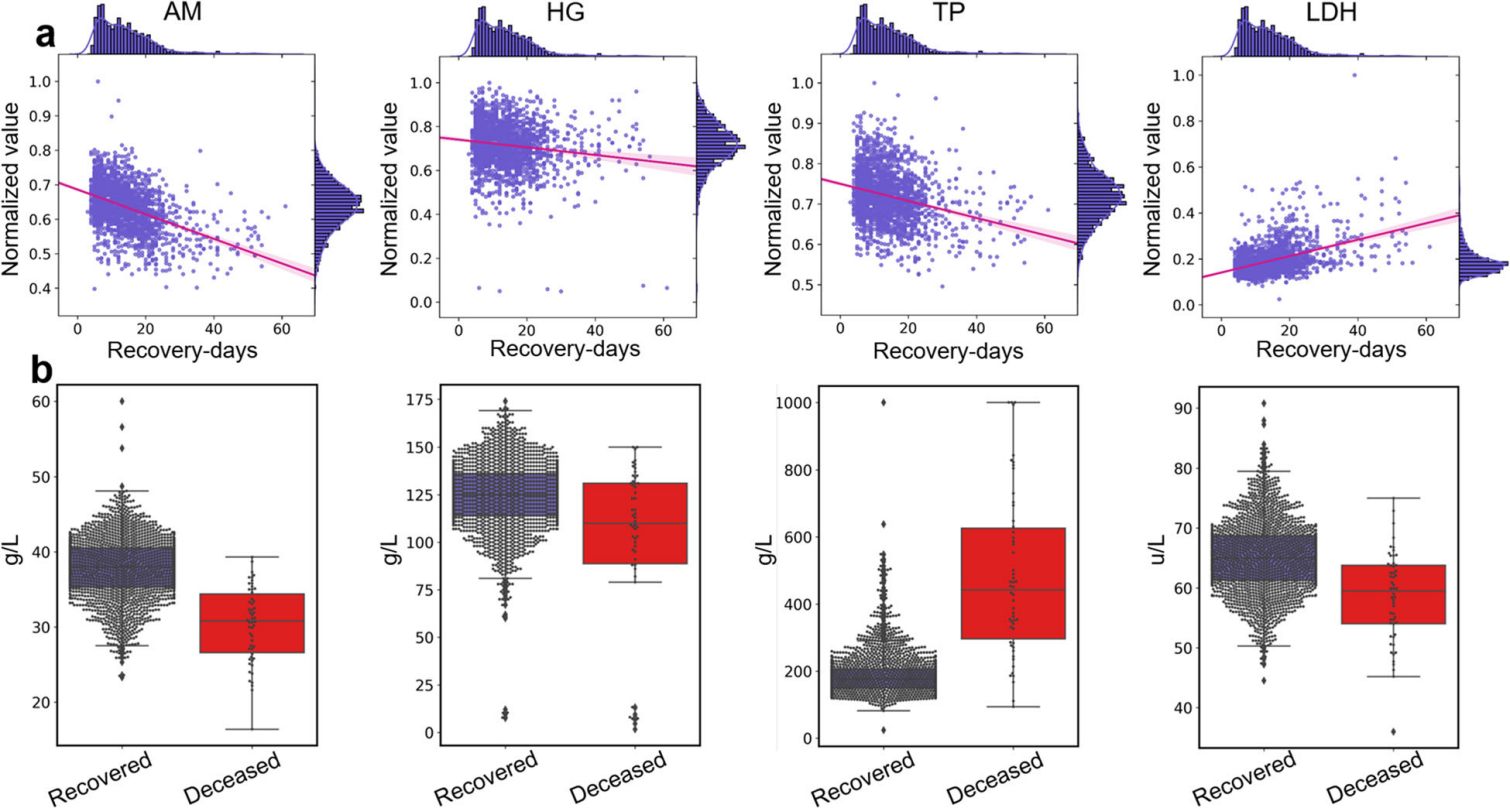
Value distribution of the main biomarkers among all recovered and deceased patients. **a** Plots of the distribution of *AM* (albumin), *HG* (hemoglobin), *TP* (total protein), and *LDH* (lactate dehydrogenase) in recovered patients, demonstrating that the recovery day is statistically negatively correlated with *AM*, *HG*, and *TP* but positively correlated with *LDH* (see the red lines, *p* value<0.001 with a Pearson correlation analysis). **b** The difference in the *AM*, *HG*, *TP*, and *LDH* statistics between the recovered and deceased patients. Compared with the recovered patients, the patients who died generally had lower levels of *AM*, *HG*, and *LDH* and higher levels of *TP*. The centerline and the bounds of each box correspond to the median value and the interquartile range, respectively, and the whiskers mark the range of the non-outlier data.

Then, we created a heatmap of the average significance of each clinical feature and the recovery days (see Fig. 5b). The heatmap demonstrates that the biomarkers *AM*, *HG*, and *TP*, the symptoms *EPC*, *DH*, and *FV*, and the comorbidities *DB*, *ARDS*, and *SK* are important for the predictions. However, the level of significance of these features differs. For example, *AM*, *HG*, and *TP* are the top 3 significant features for the prediction of recovered patients, whereas the biomarkers *LDH*, *α-HBDH*, and comorbidities *SK*, *ARDS*, and *DB* are the most important features for the prediction of deceased patients (recovery day 32 indicates death). To further illustrate the difference, we plotted the statistical significance of the top 15 clinical features in Fig. 5c, which reveals that biomarkers (i.e., *AM*, *HG*, and *TP*), symptoms (i.e., *EPC*, *DH*, and *FV*), and comorbidities (i.e., *DB*, *ARDS*, and *SK*) are indeed important for the prediction. To verify the reliability of the result, we also performed a Pearson analysis^38^ to calculate the correlation coefficients between each feature and the recovery time. The Pearson coefficients (Fig. 5d) demonstrate that the top 15 features shown in Fig. 5c indeed are strongly correlated with the recovery time of COVID-19 patients (*p* value < 0.001, except for the discrete features *EPC*, *DH*, and *SK*). Finally, we also conducted a statistical analysis of the main biomarkers among the recovered and deceased patients. The value distribution of *AM*, *HG*, *TP*, and *LDH* is plotted in Fig. 6a, which demonstrates that the recovery day is statistically negatively correlated with *AM*, *HG*, and *TP* but positively correlated with *LDH* (see the red lines). Compared with the recovered patients, patients who died normally had lower levels of *AM*, *HG*, and *LDH* and higher levels of *TP* (Fig. 6b).

### Comparison with benchmark survival models

**Fig. 7:**
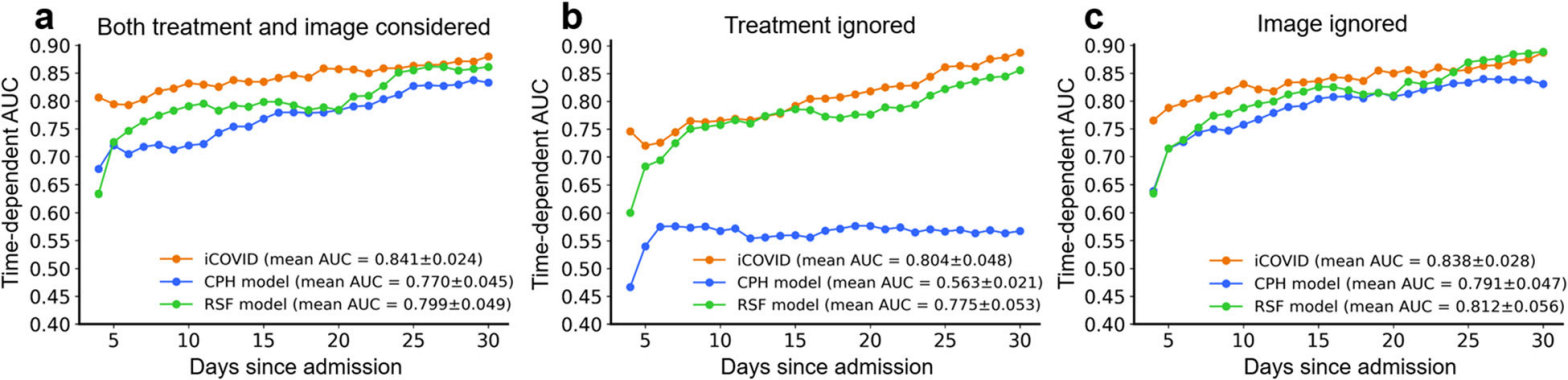
Comparison across different survival models. **a**–**c** Time-dependent AUC scores of iCOVID, Cox’s proportional hazard (CPH) model, and random survival forest (RSF) model. The curves demonstrate that the proposed iCOVID model can achieve superior performance over both the CPH and RSF models regardless of whether treatment or image are considered.

Subsequently, we compared the proposed iCOVID model with two benchmark survival models that are widely used in the field of survival analysis, including CPH^29^ model and the random survival forest (RSF)^39^ model (see Methods for the implementation details). CPH is a linear model and assumes that the “possibility” of experiencing an event remains constant over time (i.e., the proportional hazard assumption), whereas the RSF model does not have this restriction by predicting a score for every time point, which is similar to the iCOVID model (i.e., time-dependent prediction). In this study, we utilized the time-dependent AUC^40^ to validate the overall performance of all models. Figure 7 demonstrates that the iCOVID model can achieve much superior performance than both the CPH and RSF models regardless of whether treatment or image is considered. When both treatment and image information are considered, iCOVID achieves a mean AUC score of 0.841 ± 0.024, whereas the mean AUC scores of CPH and RSF are only 0.770 ± 0.045 and 0.799 ± 0.049, respectively (Fig. 7a). If treatment information is ignored (Fig. 7b), the performances of all models are worse, especially the CPH model, which obtains a mean AUC score of only 0.563 ± 0.021. However, the iCOVID and RSF models still achieve promising performance with the mean AUC scores of 0.804 ± 0.048 and 0.775 ± 0.053, respectively. When image information is ignored (Fig. 7c), the performance of the iCOVID model is slightly inferior, with a mean AUC score of 0.837 ± 0.027. Interestingly, the performances of the CPH model and the RSF model are even improved when image information is not considered.

### External validation

To evaluate the generalization ability of iCOVID, we also tested iCOVID’s performance using two additional cohorts, i.e., Taikang and Guanggu. Table 2 shows the mean and standard deviation results achieved by the five models trained with fivefold cross-validation using the Huoshenshan data set. The following three main conclusions can be drawn: (1) iCOVID can still achieve promising performance in both external data sets despite its inferior performance compared with that using the Huoshenshan data set. In particular, the TD-CI score and the MADE obtained in Guanggu cohort (71.5 ± 1.8%; 4.8 ± 4.0 days) are relatively close to those obtained in the Huoshenshan data set (76.3%; 4.4 ± 3.9 days). (2) We can observe that both TD-CI and MADE are worse when treatment schemes are ignored. This phenomenon further proves that treatment schemes indeed have a significant impact on recovery-time predictions. (3) Once CT images are ignored, iCOVID can achieve inferior but still comparative performance as demonstrated by the results corresponding to iCOVID and iCOVID without CT images.

**Table 2:** External validation results of fivefold cross-validation models.

		Taikang			Guanggu		
Models	Group	Patients	TD-CI (%)	MADE (days)	Patients	TD-CI(%)	MADE (days)
iCOVID							
	All recovered	387	69.2 ± 2.1	5.0 ± 3.4	60	71.5 ± 1.8	4.8 ± 4.0
	Low-risk	306	70.0 ± 1.7	5.0 ± 3.4	32	70.4 ± 2.7	4.4 ± 3.5
	High-risk	81	69.2 ± 2.4	4.9 ± 3.5	28	72.4 ± 0.6	5.3 ± 4.5
iCOVID w/o treatments							
	All recovered	387	63.0 ± 1.5	7.0 ± 5.3	60	54.4 ± 3.4	8.2 ± 6.2
	Low-risk	306	63.7 ± 1.0	6.9 ± 5.1	32	49.7 ± 6.3	7.3 ± 5.6
	High-risk	81	62.5 ± 2.7	7.5 ± 5.9	28	54.7 ± 3.3	9.3 ± 6.7
iCOVID w/o CT images							
	All recovered	387	69.2 ± 2.7	5.4 ± 3.7	60	72.5 ± 1.0	5.2 ± 4.2
	Low-risk	306	69.7 ± 3.0	5.4 ± 3.6	32	70.2 ± 2.3	4.7 ± 3.6
	High-risk	81	68.9 ± 2.9	5.1 ± 3.7	28	71.9 ± 2.8	5.8 ± 4.8

*w/o* without, *low-risk* mild and moderate, *high-risk* severe and critical.

## Discussion

In this paper, we proposed a deep learning-based time-to-event analysis framework named iCOVID that can successfully achieve early recovery-time prediction of COVID-19 patients at admission within 48 hours. Extensive experiments and statistical analysis of multicenter data demonstrated that the average error between the predicted and true recovery days was ~4.5 days (see Table 1). Most importantly, we investigated a large number of clinical features as listed in Supplementary Table 1 that might be relevant for the prediction, including demographics (age and gender), symptoms, comorbidities, and biomarkers. Our experimental results revealed that albumin, hemoglobin, total protein (*TP*), expectoration, diarrhea, soreness, fever, cough, diabetes, *ARDS*, and shock were highly related to the recovery-time prediction (see Fig. 5b), which was consistent with prior studies^41,42^. In addition to the above-mentioned features, we also investigated the impact of treatment schemes on the predictions, which has not been considered in most previous studies to the best of our knowledge. Our experimental results demonstrated that treatment schemes were indeed significant in the prediction of the recovery time (see Table 1 and Figs. 4, 7). Since iCOVID considers treatment schemes, it can be integrated with a computer-aided diagnosis system of COVID-19 to help clinicians determine the optimal treatment from various predefined treatment schemes, which can reduce patients’ recovery time to the greatest extent.

The results shown in Fig. 7 demonstrate that the proposed deep learning method can achieve much superior performance over the benchmark CPH model and the RSF model. A previous study^43^ demonstrated that the CPH model normally has limited performance owing to the proportional hazard assumption, and the RSF model is more suitable for complicated applications as it can build a nonlinear relationship between the variables and outcomes. However, RSF is a nondifferentiable model and is widely recognized in processing discrete variables (e.g., the symptoms and comorbidities investigated in this study). In contrast, the deep learning method is more adaptive to both discrete and continuous variables (e.g., biomarkers). In addition, treatment schemes and baseline clinical features are much more significant than CT images for the prediction, regardless of model type, further revealing that the recovery of patients is more relative to precisely individualized treatment schemes. Furthermore, the external validation demonstrates that iCOVID has promising generalization ability, even though the performance is inevitably reduced (see Table 2) owing to the variation of data distribution across the multi-site data sets (see Supplementary Tables 1–3).

We reviewed representative studies concerning computer-aided COVID-19 prognosis (see Supplementary Table 5). Most studies^17,19,20,44–46^ focused on developing deep learning or machine learning classifiers for risk prediction (severity or mortality) of COVID-19 patients. The iCOVID model developed in our study can also be applied for risk assessment of COVID-19 patients as illustrated in Fig. 3c, d. For example, if a patient is predicted to recover after a long time (e.g., 10 days), he/she should be triaged as high-risk^20^. In addition, we considered deceased patients and assumed that their recovery day was 32 in the model development, which allowed us to screen patients at a high risk of death. These patients can be easily identified by observing the curve shape as illustrated by patient #4 in Fig. 3d. These patients should have a flat curve with a peak on the last day of the time range. To validate the performance of iCOVID in identifying deaths, we drew ROC curves (see Supplementary Fig. 3) and calculated the AUC scores based on the predicted probability *P*_*T*_ of all patients. The AUC scores obtained using the Huoshenshan, Taikang, and Guanggu data sets were 94.8 ± 3.3%, 94.4 ± 2.6%, and 73.6 ± 8.3%, respectively. Data imbalance damages the AUC score obtained using the Guanggu data set, because only two deaths suffered from shock (2 of 20 deaths), which is one of the most important features for the identification of deaths (see Fig. 5b). Thus, some deaths in the Guanggu data set might be treated by iCOVID as patients who have a high probability of recovering. Promisingly, iCOVID still considers these patients at a high-risk level, and the average predicted recovery time of these patients is as high as 25.8 ± 6.5 days. It is not informative that predicting poor outcomes for patients suffering from shock or ARDS. However, ~50% of deceased patients investigated in this study were shock-free and ARDS-free within 48 hours after admission (see Supplementary Table 3). The AUC scores demonstrate that iCOVID can also precisely identify these high-risk patients.

We found only two studies that were related to recovery-time prediction. Yue et al.^47^ trained a random forest model to classify patients with different hospital stay using 1218 radiomic features^48^ extracted from CT images, whereas Liu et al.^49^ used a Kaplan–Meier analysis^50^ to determine the risk factors associated with the length of hospital stay. However, these studies had the following two main drawbacks: (1) a shortage of data for model development (<100 patients) and (2) a modeling method that was too simplistic for complicated clinical scenes, such as using heterogeneous multimodal data to predict the length of hospital stay. In particular, the Kaplan–Meier method can only be used for univariate analysis. In contrast, the proposed iCOVID solution is more practical. During the training stage, iCOVID can fully use data from patients with different outcomes (i.e., recovered, deceased, and censored) to learn the time-variant nonlinear relationship between multimodal information and events. Then, during the testing stage, iCOVID can directly estimate the length of days the patient needs to recover. Furthermore, knowledge of clinical factors, especially biomarkers that are highly correlated with the recovery time of COVID-19 patients, is also clinically important. For this purpose, an FSR mechanism is designed and incorporated with the model as a subnetwork to learn the feature significance, allowing us to screen the most important clinical features and provide strong individual interpretability of the prediction (see Fig. 3e) rather than simply statistical interpretability.

Notably, the future work includes the following limitations that are planned to be addressed. First, the model was developed and evaluated using data collected only from three hospitals and the number of patients for the external validation is relatively small. To further validate the clinical application value of the iCOVID model, we plan to perform prospective validation with cooperative hospitals. Second, the iCOVID model uses a variety of information (i.e., treatment schemes, CT images, and clinical features) as input to make predictions. However, it might be difficult to simultaneously collect all information in clinical practice. Therefore, we also validated the performance of iCOVID fed with only baseline features (all 46, top 20, top 15, top 10, and top 5 clinical features). The results demonstrate that iCOVID can still achieve promising performance, especially when considering only the top 20 and top 15 features, respectively, which can be normally obtained within 48 hours after admission (Supplementary Fig. 4). Third, the proposed FSR mechanism can reveal the significance of each clinical feature but cannot reflect the positive or negative correlation between the features and the recovery time as demonstrated by the red lines in Fig. 6. Therefore, the FSR mechanism should be further improved in future work to enhance its applicability in clinical practice. Finally, our experiments demonstrated that treatment schemes have a significant impact on the model performance. However, a more comprehensive investigation of the relationship between treatments and the recovery of COVID-19 patients needs to be performed.

In conclusion, we developed a deep learning-based time-dependent prognostic analysis framework that is applicable for the early recovery-time prediction of COVID-19 patients. We demonstrated that considering both treatment schemes and patient covariates (i.e., CT scans, demographics, symptoms, comorbidities, and biomarkers) for model development can significantly improve the prediction performance. The proposed work is not only vital for the study of COVID-19 pneumonia but also universally significant for the early prognostic prediction of other respiratory infectious diseases, especially viral pneumonia.

## Methods

### Ethics

The study was approved by the Ethics Committee of the First Affiliated Hospital of Army Medical University with approval number KY2020277, and the study was performed according to the principles of the Declaration of Helsinki. Since it is a retrospective study and presents no more than minimal risk, a waiver for informed consent was granted by the Ethics Committee.

### Materials

We collected the information of 2530 COVID-19 patients from Huoshenshan Hospital, which was built temporarily for the emergency treatment of patients in Wuhan, China. We also collected the information of 398 and 80 patients from Taikang Tongji (Wuhan) Hospital and Maternity and Child Healthcare Hospital (Guanggu) in Wuhan, respectively. SARS-CoV-2 infection was confirmed by reverse transcription polymerase chain reaction (RT-PCR) among all patients between 1 February and 31 March 2020. All specimens were extracted from nasal and throat swabs using the same standardized protocol. Confirmed cases of COVID-19 were defined as positive RT-PCR according to World Health Organization interim guidance^51^. Strict recovery criteria were executed according to the diagnostic and treatment guideline for COVID-19 issued by the Chinese National Health Committee (version seventh)^52^. All the following recovery criteria had to be met for hospital discharge or discontinuation of quarantine: (1) normal temperature lasting longer than 3 days, (2) resolved respiratory symptoms, (3) substantially improved acute exudative lesions on chest CT images, and (4) two consecutively negative RT-PCR test results separately by at least 1 day. As summarized in Supplementary Table 1, the patient information included age, gender, symptoms, comorbidities, and biomarkers, which were acquired within 2 days of the patients’ admission to the hospitals (average 0.58 ± 1.52 days). The COVID-19 severity level, the number of days of hospital stay, the treatment type, and the outcome (censored data, recovery, or death) of each patient were also collected to build the survival analysis data set. The outcome-time since admission was as follows: recovery:14.5 ± 8.2 days (minimum/maximum: 3/61 days) and death: 13.5 ± 9.6 days (minimum/maximum: 3/50 days). All patients with censored data were lost to follow-up as they were transferred to other hospitals, and it is only known that these patients were in a remission state on the date of transfer. Finally, many previous studies^53–56^ have reported that CT images can provide vital clues for prognostic estimation. Therefore, we also collected the primitive CT scan of each patient following admission within 48 hours. However, we only selected scans that were reconstructed with a slice thickness of ≤3 mm. Owing to these selection criteria and other unknown reasons, the imaging data of a total of 1492 patients were not considered during the data collection process. Thus, only 1516 patients (mild and moderate: 922; severe and critical: 594) had CT scan information in our data set.

### Data preprocessing

Different features have different magnitude ranges. For example, the lymphocytic absolute value is generally lower than 5.0/L, whereas the value of TP is usually larger than 50 g/L. We found that the framework performance can be adversely affected if the framework is trained with inputs of the original feature values. In particular, it is difficult for the FSR mechanism to learn regression coefficients that precisely reflect the significance of each feature. To reduce this impact, we normalized all features to ensure that their values ranged between 0 and 1 before feeding them into the framework (missing values were set to 0 by default). Regarding the CT scans, we first resized the scans to the same voxel size of 1 mm ×1 mm × 1 mm using bilinear interpolation to reduce variation across different scans, especially the slice thickness. Subsequently, we obtained the lung region mask of each scan using 3D-Unet trained for lung region segmentation from chest CT images^2^. This mask was consequently used to calculate a hull convex region for cropping a refined lung-only CT scan intended to remove image noise outside the lungs and reducing the cost of GPU memory. All cropped subvolumes were downsampled to the same size of 48 × 48 × 48 before feeding to the framework due to memory limitations and computational efficiency.

### Network details

The network architecture of the framework is shown in Fig. 3a. The inputs to the framework were the treatment scheme (a 19-dimensional vector), lung CT images sized 48 × 48 × 48, and clinical features (age, gender, symptoms, comorbidities, and biomarkers) represented by a 46-dimensional vector. The convolutional neural network (CNN) VGG-16^30^ was modified to a 3D version and used to extract a 128-dimensional imaging feature vector from the lung CT images. Subsequently, the 46-dimensional feature vector was fed to the FSR module to generate a weighted feature vector. This vector was finally combined with the 128-dimensional imaging feature vector and the 19-dimensional treatment scheme vector using cascaded fully-connected layers (i.e., the hidden layers in Fig. 3a) for the prognostic estimation of the number of days a patient needs to recover. In our implementation, the hidden layers were composed of four fully connected layers with 256, 512, 512, and 256 neurons. A rectified linear unit was empirically selected as the activation function, and dropout^57^ was applied after each fully connected layer during the training stage to avoid overfitting.

### FSR mechanism

The FSR mechanism is designed as a subnetwork to learn a 46-dimensional coefficient vector with each element representing the significance of each feature in the 46-dimensional feature vector, which intuitively provides interpretability to the prediction result. For example, by sorting the learned coefficients, we can determine which features make the greatest contribution to the prediction. Formally, let the feature vector and coefficient vector be represented by ${\overrightarrow {\boldsymbol{x}}} = [x_1,x_2, \ldots ,x_K]$ and ${\overrightarrow {\boldsymbol{\omega}}} = [\omega _1,\omega _2, \ldots ,\omega _K]$ (*K* = 46), respectively, and the aim is to generate a weighted feature vector ${\overrightarrow {\boldsymbol{x}}} ^\prime = [\omega _1x_1,\omega _2x_2, \ldots ,\omega _Kx_K]$ that is finally fed to the hidden layers for the prediction. Each coefficient *ω*_*k*_ in the vector ${\overrightarrow {\boldsymbol{\omega}}}$ is obtained by the softmax function as follows:

1 $$\omega _k = \frac{{{{{\mathrm{exp}}}}\left\{ {f\left( {\left. {\overrightarrow {\boldsymbol{x}} } \right|\theta } \right)_k} \right\}}}{{\mathop {\sum }\nolimits_{i = 1}^K {{{\mathrm{exp}}}}\left\{ {f\left( {\left. {\overrightarrow {\boldsymbol{x}} } \right|\theta } \right)_i} \right\}}},$$

where *f*(·) indicates a subnetwork with trainable parameters *θ*. Since the weighting coefficients are calculated using the softmax function, they are subject to $\mathop {\sum }\nolimits_{k = 1}^K \omega _k = 1$. Intuitively, the FSR module can be simply implemented by cascading fully connected layers with each layer followed by an activation layer (e.g., SeLU^58^). The final fully connected layer consists of *K* neurons that are connected to a softmax layer to produce the weighting coefficients. Notably, the fully connected layer must be initialized with 1.0 to guarantee that all features have an identical impact at the beginning of training.

### Multi-event loss function

The network was trained by minimizing a multi-event loss, comprising the following five parts:

2 $${{{\mathcal{L}}}} = \lambda _1{{{\mathcal{L}}}}_{{{{\mathrm{censor}}}}} + \lambda _2{{{\mathcal{L}}}}_{{{{\mathrm{recover}}}}} + \lambda _3{{{\mathcal{L}}}}_{{{{\mathrm{rank}}}}} + \lambda _4{{{\mathcal{L}}}}_{{{{\mathrm{death}}}}} + \lambda _5\left\| {\overrightarrow {\boldsymbol{\omega}} } \right\|_1,$$

where ${{{\mathcal{L}}}}_{{{{\mathrm{censor}}}}}$, ${{{\mathcal{L}}}}_{{{{\mathrm{recover}}}}}$, and ${{{\mathcal{L}}}}_{{{{\mathrm{death}}}}}$ indicate the loss for handling censored data, recoveries, and deaths in the prognostic estimation task, respectively. ${{{\mathcal{L}}}}_{{{{\mathrm{rank}}}}}$ is a raking loss that is applied to the recoveries to address the time-variant issue. The ranking loss adapts the idea of concordance^59^ as follows: a patient who recovered on day t_*_ should have a higher probability of recovering on day t_*_ than any patient who did not yet recover on day t_*_. The last term $\left\| {\overrightarrow {\boldsymbol{\omega}} } \right\|_1$ is the L1-norm, which helps learn the sparse coefficient vector. *λ*_1_, *λ*_2_, *λ*_3_, *λ*_4_, and *λ*_5_ are hyperparameters used to control the contribution of each term in Eq. 2. These hyperparameters are empirically set to 1, 2, 1, 5, and 1. The details are further explained as follows:

- Loss ${{{\mathcal{L}}}}_{{{{\mathrm{censor}}}}}$ is defined as follows:
  3 $${{{\mathcal{L}}}}_{{{{\mathrm{censor}}}}} = - \frac{1}{{N_{{{{\mathrm{censor}}}}}}}\mathop {\sum }\limits_{n = 1}^N \left\{ {{{{\mathrm{sgn}}}}\left( {\sigma _n = 0} \right) \cdot \log \left[ {1 - F\left( {t_n\left| {\overrightarrow {\boldsymbol{x}} _n} \right.,{{{\boldsymbol{I}}}}_n,\overrightarrow {\boldsymbol{\tau}} _n} \right)} \right]} \right\},$$
  where sgn(·) denotes an indicator function. *N*_censor_ indicates the number of censored patients in the minibatch with size *N*. *F*(*) is the CIF, which is defined by:
  4 $$F\left( {t_n\left| {\overrightarrow {\boldsymbol{x}} _n} \right.,I_n,\overrightarrow {\boldsymbol{\tau}} _n} \right) = \overrightarrow {\boldsymbol{P}} \left( {t \le t_n\left| {\overrightarrow {\boldsymbol{x}} _n} \right.,I_n,\overrightarrow {\boldsymbol{\tau}} _n} \right) = \mathop {\sum }\limits_{n = 1}^{t_n} P\left( {t\left| {\overrightarrow {\boldsymbol{x}} _n} \right.,I_n,{\overrightarrow {\boldsymbol{\tau}} _n}} \right),$$
  where ${\overrightarrow {\boldsymbol{P}}} ( \ast )$ is the estimated probability distribution. The target of Eq. 3 minimizes all probabilities $\left\{ {P_1,P_2, \ldots ,P_{t_n}} \right\}$ based on the prior knowledge that each patient with censored data did not yet recover on the last recorded day *t*_*n*_.
- Loss ${{{\mathcal{L}}}}_{{{{\mathrm{recover}}}}}$ is defined as follows:
  5 $${{{\mathcal{L}}}}_{{{{\mathrm{recover}}}}} = - \frac{1}{{N_{{{{\mathrm{recover}}}}}}}\mathop {\sum }\limits_{n = 1}^N \left\{ {{{{\mathrm{sgn}}}}\left( {\sigma _n = 1} \right) \cdot \log \left[ {P(t_n\left| {\overrightarrow {\boldsymbol{x}} _n} \right.,{{{\boldsymbol{I}}}}_n,{\overrightarrow {\boldsymbol{\tau}} _n)}} \right]} \right\},$$
  where *N*_recover_ is the number of recovered patients in the minibatch. Equation 5 drives the network to learn a maximum probability on the *t*_*n*_ day when the *n*th patient recovers after admission.
- Ranking loss ${{{\mathcal{L}}}}_{{{{\mathrm{rank}}}}}$ is calculated as follows:
  6 $${{{\mathcal{L}}}}_{{{{\mathrm{rank}}}}}=\mathop {\sum}\limits_{n \ne m}\bigg\{{{{\mathrm{sgn}}}}(t_n\,<\,t_m)\cdot {{{\mathrm{exp}}}}\bigg[\frac{{F\left({t_n\left|{\overrightarrow {\boldsymbol{x}}_m}\right.,{{{\boldsymbol{I}}}}_m,\overrightarrow{\boldsymbol{\tau}}_m} \right)-F\left({t_n\left| {\overrightarrow{\boldsymbol{x}} _n} \right.,{{{\boldsymbol{I}}}}_n,\overrightarrow {\boldsymbol{\tau}} _n} \right)}}{\alpha }\bigg]\bigg\} ,$$
  where *α* denotes a hyperparameter that is empirically set to 0.2 in this study. Since exp(*) is a convex function, minimizing Eq. 6 equals maximizing the distance between $F\left( {t_n\left| {\overrightarrow {\boldsymbol{x}} _n} \right.,{{{\boldsymbol{I}}}}_n,\overrightarrow {\boldsymbol{\tau}} _n} \right)$ and $F\left( {t_n\left| {\overrightarrow {\boldsymbol{x}} _n} \right.,{{{\boldsymbol{I}}}}_m,\overrightarrow {\boldsymbol{\tau}} _m} \right)$ subject to $F\left( {t_n\left| {\overrightarrow {\boldsymbol{x}} _n} \right.,{{{\boldsymbol{I}}}}_n,\overrightarrow {\boldsymbol{\tau}} _n} \right) > F\left( {t_n\left| {\overrightarrow {\boldsymbol{x}} _m} \right.,{{{\boldsymbol{I}}}}_m,\overrightarrow {\boldsymbol{\tau}} _m} \right)$.
- Loss ${{{\mathcal{L}}}}_{{{{\mathrm{death}}}}}$ is defined as follows:
  7 $${{{\mathcal{L}}}}_{{{{\mathrm{death}}}}} = - \frac{1}{{N_{{{{\mathrm{death}}}}}}}\mathop {\sum }\limits_{n = 1}^N \left\{ {{{{\mathrm{sgn}}}}\left( {\sigma _n = 2} \right) \cdot \log \left[ {1 - F\left( {T - 1\left| {\overrightarrow {\boldsymbol{x}} _n} \right.,{{{\boldsymbol{I}}}}_n,\overrightarrow {\boldsymbol{\tau}} _n} \right)} \right]} \right\},$$
  where *N*_death_ is the number of deceased patients in the minibatch, and *T* is the last day in the estimation time range (*T* = 32 in this study). The target of Eq. 7 minimizes all probabilities {$P_1,P_2, \ldots ,P_{T - 1}$} and maximizes *P*_*T*_ corresponding to each deceased patient. We can observe that Eq. 7 is similar to Eq. 3. According to this definition, deceased patients are treated as a special type of patients with censored data.

### Evaluation metrics

The TD-CI and the MADE were calculated to evaluate the performance of the recovery event. Given the CIF in Eq. 4, the TD-CI *C*^td^is defined as follows:

8 $$C^{{{{\mathrm{td}}}}} = \frac{{\mathop {\sum }\nolimits_{n \ne m} {{{\mathrm{sgn}}}}(t_n\, < \,t_m) \cdot {{{\mathrm{sgn}}}}(F\left( {t_n\left| {\overrightarrow {\boldsymbol{x}} _n} \right.,{{{\boldsymbol{I}}}}_n,\overrightarrow {\boldsymbol{\tau}} _n} \right) > F\left( {t_n\left| {\overrightarrow {\boldsymbol{x}} _n} \right.,{{{\boldsymbol{I}}}}_m,\overrightarrow {\boldsymbol{\tau}} _m} \right))}}{{\mathop {\sum }\nolimits_{n \ne m} {{{\mathrm{sgn}}}}(t_n\, < \,t_m)}},$$

which counts the number of predictions that correctly abide by the idea of concordance^59^. The MADE *d*^ma^ is calculated as follows:

9 $$d^{{{{\mathrm{ma}}}}} = \frac{1}{N}\mathop {\sum }\limits_{n = 1}^N \left| {t_n - {{{\mathrm{argmax}}}}\left( {\overrightarrow {{\boldsymbol{P}}_n} } \right)} \right|,$$

where ${\overrightarrow {{\boldsymbol{P}}_n}}$ is the predicted probability distribution of the *n*^th^ patient. For the internal validation, the performance was evaluated statistically in terms of 95% confidence interval of the above-mentioned TD-CI and MADE metrics. The 95% CI values were calculated using the bootstrap method^60^.

### Fivefold cross-validation

The five subsets $\left\{ {{{{\mathrm{Cohort}}}}\_i\left| {i = 1,2, \ldots ,5} \right.} \right\}$ of the Huoshenshan data set were used to train five independent models $\left\{ {{{{\mathrm{M}}}}_j{{{\mathrm{|}}}}j = 1,2, \ldots ,5} \right\}$for internal validation. Each model M_*j*_ was trained using four subsets $\{ {{{\mathrm{Cohort}}}}\_i|i = 1,2, \ldots ,5\,{{{\mathrm{and}}}}\,i \,\ne \,j\}$ and tested using the remaining subset.

### Ablation experiments

To validate the impact of treatments and CT images on the prediction performance, we also trained iCOVID models without considering any treatment information, i.e., setting all *τ* in the ground-truth treatment scheme to zero during the training stage, and iCOVID models without using any CT image information, i.e., setting all voxel values in the image matrix to zero during the training stage. The clinical data, i.e., demographics, symptoms, comorbidities, and biomarkers, were used as baseline information in all models.

### Implementation of benchmark models

The fivefold CPH models and the RSF models were trained using CoxnetSurvivalAnalysis and RandomSurvivalForeset (with 100 trees) implemented in the python library Scikit-survival: https://scikit-survival.readthedocs.io/en/latest/index.html. Because the CPH model and the RSF model cannot directly process CT images, we first extracted the convolutional feature vectors from the CT images using the CNN encoders of the iCOVID models, and then, we used the feature vectors as the input to the benchmark models. Similar to the aforementioned ablation study, we also trained the benchmark models without considering any treatment or image information for comparison.

### Training details

The framework was implemented using Google TensorFlow (version 2.0 with Keras API) on an NVIDIA RTX 2080Ti GPU. During the training stage, the networks were optimized by gradient descending with gradients estimated by the Adam optimizer under the constraint of minimizing the multi-event loss. The learning rate was 0.001, decaying every 100 iterations with an exponential rate of 0.96. The total number of iterations was 2k (20 epochs multiplied by 100 iterations). At each iteration, a minibatch of 72 samples was fed to the networks. We augmented the CT scans by randomly rotating each scan to 0, 90, 180, and 270 degrees, and randomly flipping the scans in the X, Y, and Z axes. For those patients without CT images, we directly input a volume of size 48 × 48 × 48 with zero values. To avoid the overfitting issue, only the model that achieved a minimum MADE using the subset applied for the online evaluation (i.e., Cohort_6, see Supplementary Fig. 1d) was saved.

### Reporting summary

Further information on research design is available in the Nature Research Reporting Summary linked to this article.

## Supplementary information

Supplementary Information

Reporting Summary

## Data Availability

The survival data sets used for modeling are not publicly available owing to privacy concerns. However, researchers can contact the corresponding author to obtain the de-identified data upon ethical approval from the Ethics Committee of Southwest Hospital, Third Military Medical University, and signature of a data usage agreement. The remaining data are available in the article and supplementary files.

## Code availability

The code, pre-trained models, and a total of 258 samples are available at: https://github.com/wangjuncongyu/covid19_recovery.
