# Supplementary material for: iCOVID: interpretable deep learning framework for early recovery-time prediction of COVID-19 patients
Source: NPJ Digit Med. 2021 Aug 16;4:124. doi: 10.1038/s41746-021-00496-3 (PMC8367981; doi:10.1038/s41746-021-00496-3)
Supplement: Supplementary file 1 — Supplementary Information [file 41746_2021_496_MOESM1_ESM.pdf]

Supplementary figures

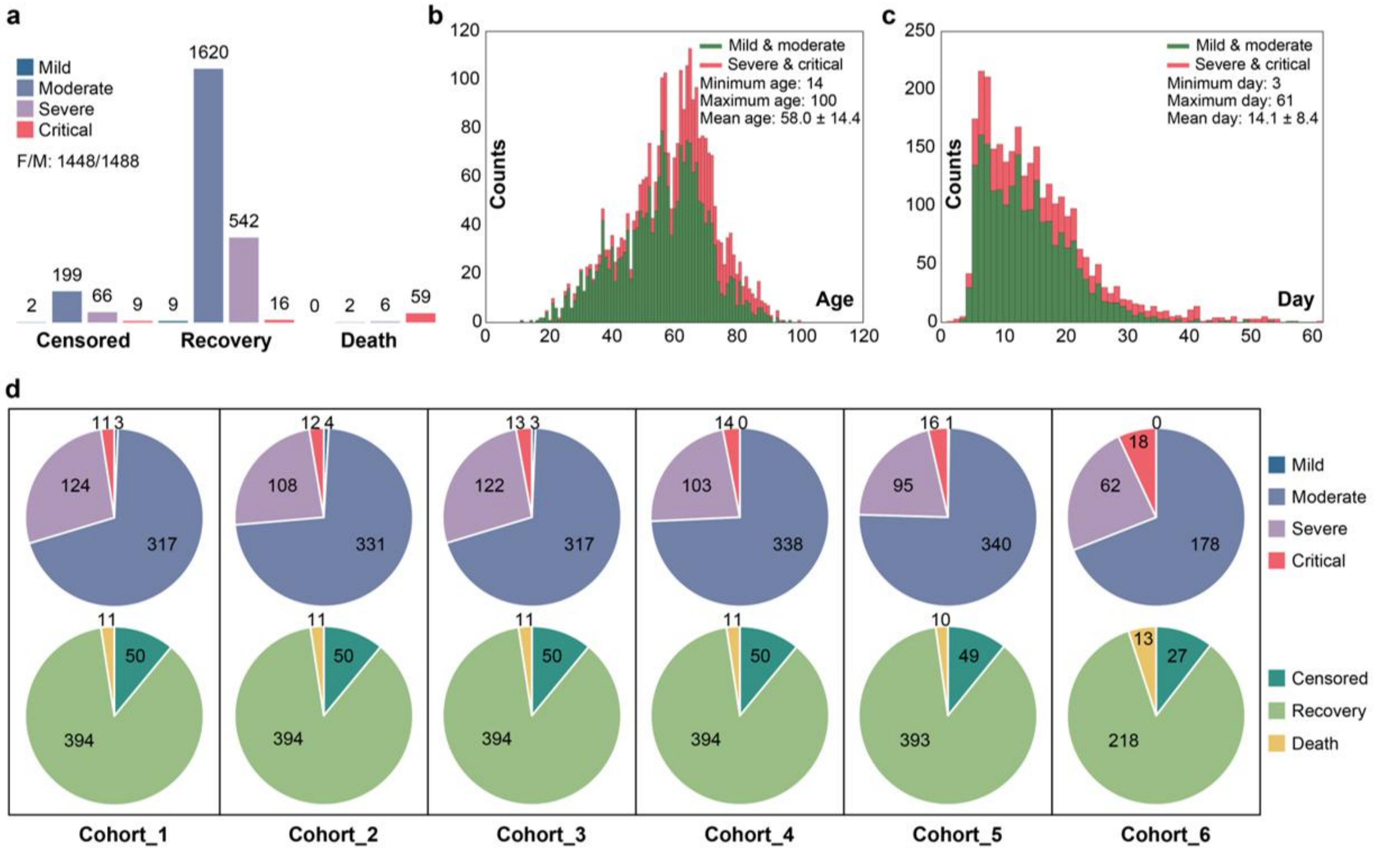

Figure 1. Patient statistics in the dataset from Huoshenshan Hospital. **a**, Number of patients with confirmed COVID-19 severity and the number of patients with censored data, recovery and death. **b**, Patient age distribution. **c**, Patient recovery day distribution. The dataset is subdivided into six cohorts, and the number of patients in each cohort is plotted in **d**. Cohort_1 to Cohort_5 are used for the five-fold cross-validation, whereas Cohort_6 is utilized for the online evaluation during the training stage.

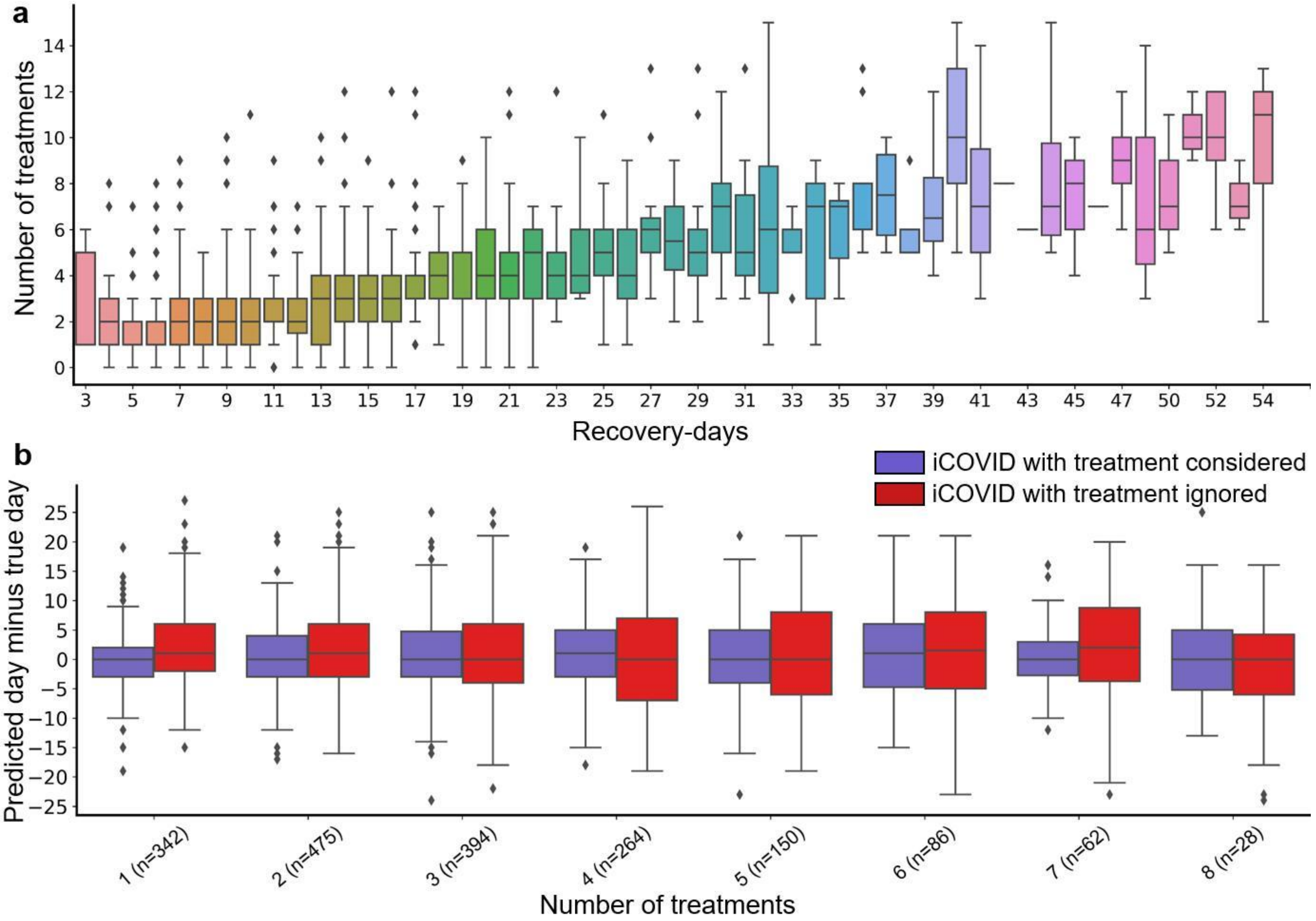

Figure 2. Treatment number distribution among patients with different recovery times and the statistics of the day error. a, Treatment number distribution demonstrates that patients who require longer days to recover normally received more treatment schemes. **b**, Statistics of day errors among different patient groups (patients in each group used the same number of treatments during their hospitalization). It can be observed that the median value and the interquartile range of the dark-blue boxes are not significantly changed with the number variation of treatments, revealing that the treatments rather than the number of treatments have a strong impact on the prediction. Besides, iCOVID can indeed achieve more stable reliable predictions by considering treatment schemes. The center line and the bounds of each box correspond to the median value and the interquartile-range, respectively, and the whiskers mark the range of the non-outlier data.

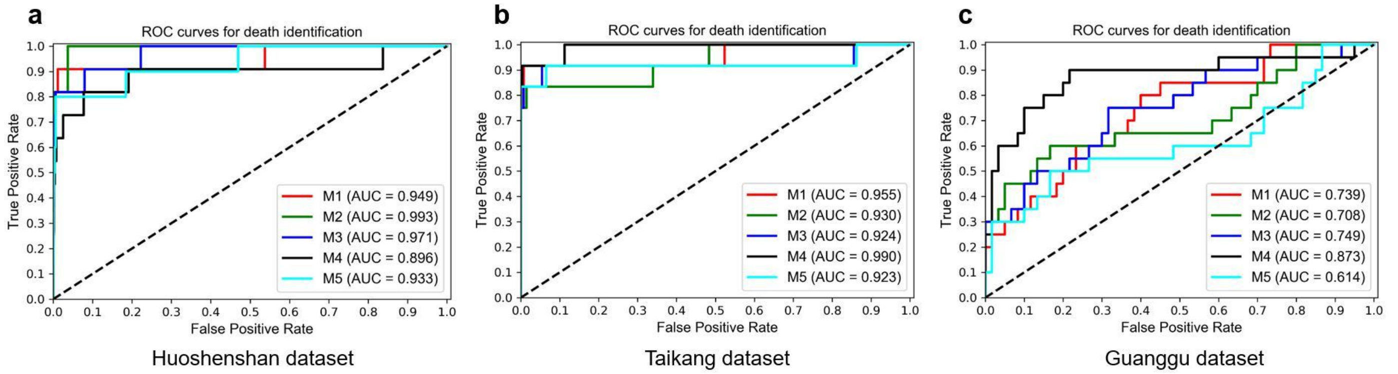

Figure 3. ROC curves for death identification. The curves demonstrate that iCOVID can also achieve promising performance in death identification using the Huoshenshan, Taikang, and Guanggu datasets. M1-M5 indicate the five models trained with five-fold cross-validation using the Huoshenshan data subsets.

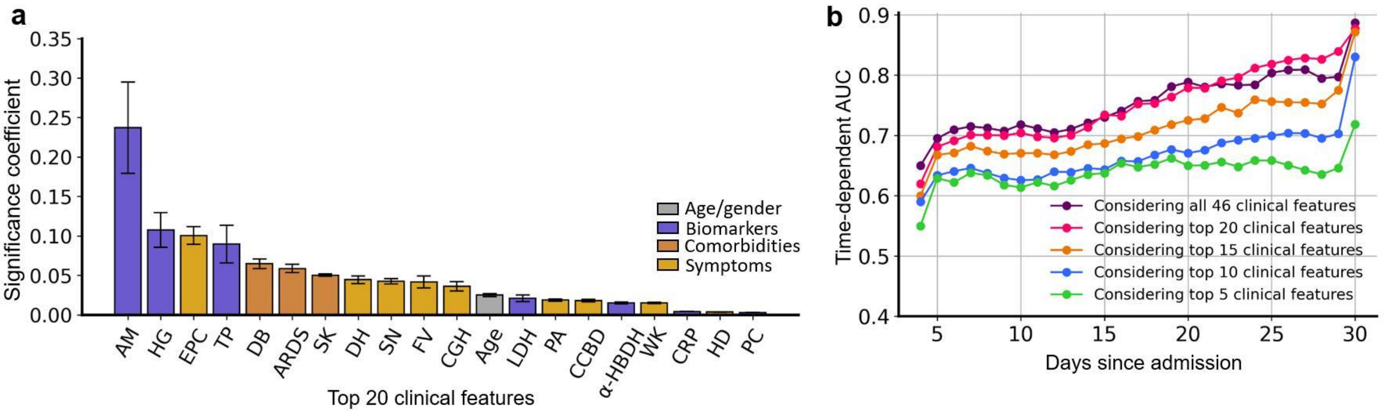

c

Clinical features	Patients	TD-CI (unit: %)	MADE (unit: days)	AUC
All 46 features	1969	71.5 (68.2-74.6)	5.8 (5.7-6.1)	0.753±0.049
Top 20 features	1969	69.5 (66.3-73.0)	6.4 (6.1-6.7)	0.752±0.061
Top 15 features	1969	66.9 (63.1-70.3)	6.1 (5.8-6.3)	0.711±0.051
Top 10 features	1969	64.4 (61.2-68.8)	6.9 (6.4-7.5)	0.667±0.043
Top 5 features	1969	63.1 (59.8-67.3)	8.0 (7.7-8.3)	0.640±0.027
(-): 95% confident interval				

Figure 4. Performance of the iCOVID model with respect to different numbers of clinical features. **a**, Average significance of the top 20 clinical features, i.e., AM: albumin; HG: hemoglobin; EPC: expectoration; TP: total protein; DB: diabetes; ARDS: acute respiratory distress syndrome; SK: shock; DH: diarrhea; SN: soreness; FV: fever; CGH: cough; LDH: lactate dehydrogenase; PA: poor appetite; CCBD: chest congestion/breathing difficulty; α-HBDH: alpha-hydroxybutyrate dehydrogenase; WK: weakness; CRP: C-reactive protein; HD: headache; PC: platelet count. **b** and **d**, Time-dependent AUC scores and metrics corresponding to iCOVID model considering all 46, top 20, top 15, top 10, and top 5 clinical features.

Supplementary tables

Table 1. Patients information and statistics in each dataset

Name	Abbr.	Normal range/value	Huoshenshan (n=2530)	Taikang (n=398)	Guanggu (n=80)
		Demographics/Images			
**Age***	Age	10~120 years old	58.0±14.4	59.6±15.7	73.1±13.8
Gender	Gender	Male/Female	M/F:1299/1231	M/F:171/27	M/F:49/31
CT scans	CT	Thickness≤3 mm	1319/52%	197/49.5%	0/0%
		Symptoms			
**Cough***	CGH	No	1864/73.4%	234/58.8%	49/61.3%
**Chest Congestion/Breathing Difficulty***	CCBD	No	1002/39.6%	134/33.7%	31/38.8%
**Diarrhea***	DH	No	170/6.7%	39/9.8%	4/5.0%
**Expectoration***	EPC	No	426/16.8%	59/14.8%	25/31.3%
**Fever***	FV	No	1852/73.2%	249/62.6%	48/58.8%
Headache	HD	No	107/4.2%	17/4.3%	4/5.0%
Nausea/Vomiting	NV	No	95/3.8%	20/5.0%	7/8.8%

**Poor Appetite***	PA	No	1536/60.7%	40/10.1%	47/58.8%
**Soreness***	SN	No	735/29.1%	26/6.5%	13/16.3%
Weakness	WK	No	1435/56.7%	151/37.9%	37/46.3%
Comorbidities					
**Acute Respiratory Distress Syndrome***	ARDS	No	45/1.8%	13/3.3%	1/1.3%
Chronic Hepatitis B	CHB	No	44/1.7%	11/2.8%	0/0.0%
**Diabetes***	DB	No	342/13.5%	46/11.6%	17/21.3%
Hypothyroidism	HPD	No	29/1.1%	4/1.0%	0/0.0%
Kidney Disease	KD	No	29/1.1%	8/2.0%	0/0.0%
Malignant Tumor	MT	No	50/2.0%	3/0.8%	6/7.5%
**Shock***	SK	No	37/1.5%	8/2.0%	2/2.5%
Biomarkers					
**Albumin***	AM	>35 g/L	604/23.9%	99/24.9%	46/57.5%
Alanine Aminotransferase	ALT	Female, ≤35 u/L; Male, ≤41 u/L	598/23.6%	82/20.6%	20/25.0%
Aspartate Aminotransferase	AST	Female, ≤32 u/L; Male, ≤40 u/L	316/12.5%	23/5.8%	18/22.5%
Alpha-hydroxybutyrate Dehydrogenase	α-HBDH	90~182 u/L	552/21.8%	67/16.8%	0/0.0%
Activated Partial Thromboplastin Time	APTT	<42 s	19/0.8%	10/2.5%	8/10.0%
Blood Glucose	BGLU	Empty stomach, 3.9~6.1 mmol/L	538/21.3%	81/20.1%	38/47.5%
C-reactive Protein	CRP	<1 mg/L	1708/67.5%	113/28.4%	71/88.8%
Creatinine	CRT	<104 umol/L	106/4.2%	7/1.8%	12/15.0%
Creatine Kinase	CK	18~198 u/L	106/5.1%	9/2.3%	7/8.8%
Creatine Kinase Isoenzyme MB Form	CK-MB	<18 u/L	157/6.2%	11/2.8%	3/3.8%
Direct Bilirubin	DBIL	<6.8 umol/L	177/7.0%	15/3.8%	27/33.8%
D-dimer	DD	<0.5 mg/L	848/33.5%	28/7.0%	55/68.8%
Fibrinogen	FIB	2~4 g/L	216/8.5%	54/13.6%	50/62.5%
**Hemoglobin***	HG	Female, ≥115 g/L; Male, ≥130 g/L	1020/40.3%	187/47.0%	41/51.3%
Interleukin-6	IL-6	108.85±41.48 ng/mL	990/39.1%	260/65.3%	52/65.0%
**Lactate Dehydrogenase***	LDH	Female, <214 u/L; Male, <255 u/L	570/22.5%	63/15.8%	23/28.8%
Lymphocytic Absolute Value	LAV	$0.8\sim3.5\times10^9$/L	275/10.9%	38/9.5%	26/32.5%
Neutrophils Absolute Value	NAV	$2\sim7\times10^9$/L	369/14.6%	61/15.3%	27/33.8%
Platelet Count	PC	$100\sim300\times10^9$/L	501/19.8%	66/16.6%	19/23.8%
Procalcitonin	PCT	<0.5 ug/L	43/1.7%	13/3.3%	11/13.8%
Prothrombin Time	PT	11~13 s	925/36.6%	75/18.8%	28/35.0%
Red Blood Cell	RBC	Female, $3.5\sim5\times10^{12}$/L;	0/0.0%	0/0.0%	0/0.0%

		Male: 4~5.5×10^{12}/L			
Thrombin Time	TT	16~18 s	1649/65.2%	151/37.9%	57/71.3%
**Total Protein***	TP	60~80 g/L	533/21.1%	54/13.6%	17/21.3%
Total Bilirubin	TB	3.4~17.1 umol/L	229/9.1%	49/12.3%	17/21.3%
Urea Nitrogen	UN	2.9~7.5 mmol/L	405/16.0%	42/10.6%	31/38.8%
White Blood Cell	WBC	4~10×10^9/L	411/16.2%	68/17.1%	24/30.0%

The top 15 features are shown in bold with asterisk; the right three columns show the number of abnormal patients

Table 2. Treatments/drugs investigated in this study and statistics in each dataset

Name	Abbr.	Category	Huoshenshan (n=2530)	Taikang (n=398)	Guanggu (n=80)
Arbidol	ABD	Antiviral drugs	1097/43.4%	257/64.6%	4/5.0%
Ribavirin	RV	Antiviral drugs	98/3.9%	51/12.8%	0/0.0%
Oseltamivir	OV	Antiviral drugs	221/8.7%	76/19.1%	16/20.0%
Piperacillin	PPL	Antibacterial drugs	32/1.3%	4/1.0%	5/6.3%
Cephalosporins	CPP	Antibacterial drugs	203/8.0%	45/11.3%	24/30.0%
Levofloxacin	LFN	Antibacterial drugs	133/5.3%	28/7.0%	1/1.3%
Linezolid	LZD	Antibacterial drugs	42/1.7%	3/0.8%	4/5.0%
Moxifloxacin	MFN	Antibacterial drugs	665/26.3%	131/32.9%	45/56.3%
Lianhua Qingwen	LQC	Chinese medicine	1452/57.4%	246/61.8%	43/53.8%
Xuebijing	XBJ	Chinese medicine	162/6.4%	21/5.3%	9/11.3%
Convalescent Plasma	CP	Immunotherapy drugs	78/3.1%	0/0.0%	9/11.3%
Immunoglobulin	IGN	Immunotherapy drugs	137/5.4%	16/4.0%	10/12.5%
Tocilizumab	TB	Immunotherapy drugs	77/3.0%	0/0.0%	44/55%
Ambroxol	ABX	Apophlegmatisant	181/7.2%	108/27.1%	42/52.5%
Acetylcysteine	CAN	Apophlegmatisant	476/18.8%	86/21.6%	15/18.8%
Heparin	HPN	Anticoagulant therapy	156/6.2%	16/4.0%	14/17.5%
Methylprednisolone	MPN	Hormone therapy	284/11.2%	11/2.8%	22/27.5%
High-flow Nasal Cannula Oxygen	HFNC	Respiratory assistance	1708/67.5%	58/14.6%	45/56.3%
Vitamin C	VC	Others	702/27.7%	238/59.8%	48/60.0%

Table 3. Number of deceased patients suffered from Shock and ARDS

Deceased patients	Huoshenshan (n=67)	Taikang (n=11)	Guanggu (n=20)
With Shock	36 (53.7%)	8 (72.3%)	2 (10.0%)
With ARDS	35 (52.2%)	11 (100.0%)	1 (5.0%)
With Shock and ARDS	22 (32.8%)	8 (72.7%)	0 (0.0%)

Number of deceased patients with Shock or ARDS (Percentage)

Table 4. Five-fold cross-validation results using the Huoshenshan dataset with different CNNs

CNN encoder	Patients	iCOVID TD-CI (unit: %)	iCOVID MADE (unit: days)	iCOVID w/o CT images TD-CI (unit: %)	iCOVID w/o CT images MADE (unit: days)
VGG-16[1]	1969	74.9 (73.6-76.3)	**4.4** (4.2-4.6)	**74.6** (72.8-76.1)	4.7 (4.5-4.8)
ResNet-34[2]	1969	**75.2** (73.9-76.5)	4.7 (4.6-4.9)	73.8 (72.7 -75.0)	4.9 (4.7-5.0)
MobileNet-v3[3]	1969	73.9 (72.1-75.3)	4.7 (4.5-4.9)	73.2 (70.5-76.5)	5.0 (4.7-5.5)
InceptionNet-v4[4]	1969	**75.2** (73.7-76.5)	5.2 (5.0-5.4)	73.8 (72.2-75.7)	4.7 (4.6-4.9)
EfficientNet-b3[5]	1969	74.7 (73.1-76.0)	4.7 (4.6-4.9)	74.4 (72.6-75.8)	**4.5** (4.4-4.7)

w/o: without; the best performance in each column is shown in bold

Table 5. State-of-the-art studies of computer-aided COVID-19 prognosis

Studies	Patients	Investigated factors	With treatment	Task	Individual interpretability	Method
Schwab et al. [6]	66430	D; B; S; C	No	Mortality	Yes	Nonlinear CPH
Lassau et al.[7]	1003	D; B; S; I	No	Severity	No	DL
Gao et al.[8]	2520	D; B; S; C	No	Mortality	No	ML
Barda et al.[9]	4179	D; B; C	No	Mortality	No	ML
Feng et al.[10]	298	D; B; C; I	No	Severity	No	LR
Liang et al.[11]	1590	D; B; C; I	No	Severity	No	Nonlinear CPH
Ning et al.[12]	1521	D; B; C; I	No	Mortality	No	DL
Subudhi et al.[13]	5308	D; B; C	No	Severity	No	ML
Xu et al.[14]	3024	D; B; S; C; I	No	Severity;	No	LR; ML; KM
Zhou et al.[15]	4442	D; B; C; T	Yes (17)	Severity	No	KM; LR
Razavian et al.[16]	3819	D; B;	No	Favorable outcome	No	LR; ML
Shamout et al.[17]	3661	D; B; I	No	Severity	No	ML; DL
Yue et al.[18]	58	I	No	Hospital stay	No	ML
Liu et al.[19]	99	B; C; T	Yes (2)	Hospital stay	No	KM
Proposed	3008	D; B; S; C; I; T	Yes (19)	Recovery-time	Yes	DL

D: demographics; B: biomarkers; S: symptoms; C: comorbidities; I: images; T: treatments;
DL: deep learning; ML: machine learning; LR: logistic regression; KM: Kaplan-Meier

References

1. Simonyan, K. & Zisserman, A. Very deep convolutional networks for large-scale image recognition. in *3rd International Conference on Learning Representations, ICLR 2015 - Conference Track Proceedings* (2015).
2. He, K., Zhang, X., Ren, S. & Sun, J. Deep residual learning for image recognition. in *Proceedings of the IEEE conference on computer vision and pattern recognition* 770–778 (2016).
3. Howard, A. G. *et al.* MobileNets: Efficient convolutional neural networks for mobile vision applications. *arXiv:1704.04861v1* (2017).
4. Szegedy, C., Ioffe, S., Vanhoucke, V. & Alemi, A. A. Inception-v4, inception-resnet and the impact of residual connections on learning. in *Thirty-First AAAI Conference on Artificial Intelligence* (2017).
5. Tan, M. & Le, Q. EfficientNet: Rethinking Model Scaling for Convolutional Neural Networks. in *Proceedings of the 36th International Conference on Machine Learning* (eds. Chaudhuri, K. & Salakhutdinov, R.) **97**, 6105–6114 (PMLR, 2019).
6. Schwab, P. *et al.* Real-time prediction of COVID-19 related mortality using electronic health records. *Nat. Commun.* **12**, 1058 (2021).
7. Lassau, N. *et al.* Integrating deep learning CT-scan model, biological and clinical variables to predict severity of COVID-19 patients. *Nat. Commun.* **12**, 634 (2021).
8. Gao, Y. *et al.* Machine learning based early warning system enables accurate mortality risk prediction for COVID-19. *Nat. Commun.* **11**, 1–9 (2020).
9. Barda, N. *et al.* Developing a COVID-19 mortality risk prediction model when individual-level data are not available. *Nat. Commun.* **11**, 4439 (2020).
10. Feng, Z. *et al.* Early prediction of disease progression in COVID-19 pneumonia patients with chest CT and clinical characteristics. *Nat. Commun.* **11**, 4968 (2020).
11. Liang, W. *et al.* Early triage of critically ill COVID-19 patients using deep learning. *Nat. Commun.* **11**, 1–7 (2020).
12. Ning, W. *et al.* Open resource of clinical data from patients with pneumonia for the prediction of COVID-19 outcomes via deep learning. *Nat. Biomed. Eng.* **4**, 1197–1207 (2020).
13. Subudhi, S. *et al.* Comparing machine learning algorithms for predicting ICU admission and mortality in COVID-19. *npj Digit. Med.* **4**, 87 (2021).
14. Xu, Q. *et al.* AI-based analysis of CT images for rapid triage of COVID-19 patients. *npj Digit. Med.* **4**, 75 (2021).
15. Zhou, J. *et al.* Development of a multivariable prediction model for severe COVID-19 disease: a population-based study from Hong Kong. *npj Digit. Med.* **4**, 66 (2021).
16. Razavian, N. *et al.* A validated, real-time prediction model for favorable outcomes in hospitalized COVID-19 patients. *npj Digit. Med.* **3**, 130 (2020).
17. Shamout, F. E. *et al.* An artificial intelligence system for predicting the deterioration of COVID-19 patients in the emergency department. *npj Digit. Med.* **4**, 80 (2021).

18. Yue, H. *et al.* Machine learning-based CT radiomics method for predicting hospital stay in patients with pneumonia associated with SARS-CoV-2 infection: a multicenter study. *Ann. Transl. Med. Vol 8, No 14 (July 2020) Ann. Transl. Med.* **8**, 1–7 (2020).
19. Liu, X. *et al.* Risk factors associated with disease severity and length of hospital stay in COVID-19 patients. *Journal of Infection* (2020). doi:10.1016/j.jinf.2020.04.008
