# Supplementary material for: iCOVID: interpretable deep learning framework for early recovery-time prediction of COVID-19 patients
Source: NPJ Digit Med. 2021 Aug 16;4:124. doi: 10.1038/s41746-021-00496-3 (PMC8367981; doi:10.1038/s41746-021-00496-3)
Supplement: Supplementary file 2 — Reporting Summary [file 41746_2021_496_MOESM2_ESM.pdf]

nature portfolio

Corresponding author(s): Dahong Qian

Last updated by author(s): Jul 19, 2021

Reporting Summary

Nature Portfolio wishes to improve the reproducibility of the work that we publish. This form provides structure for consistency and transparency in reporting. For further information on Nature Portfolio policies, see our Editorial Policies and the Editorial Policy Checklist.

Statistics

For all statistical analyses, confirm that the following items are present in the figure legend, table legend, main text, or Methods section.

n/a	Confirmed	
☐	☒	The exact sample size (*n*) for each experimental group/condition, given as a discrete number and unit of measurement
☐	☒	A statement on whether measurements were taken from distinct samples or whether the same sample was measured repeatedly
☐	☒	The statistical test(s) used AND whether they are one- or two-sided<br>*Only common tests should be described solely by name; describe more complex techniques in the Methods section.*
☐	☒	A description of all covariates tested
☐	☒	A description of any assumptions or corrections, such as tests of normality and adjustment for multiple comparisons
☐	☒	A full description of the statistical parameters including central tendency (e.g. means) or other basic estimates (e.g. regression coefficient) AND variation (e.g. standard deviation) or associated estimates of uncertainty (e.g. confidence intervals)
☐	☒	For null hypothesis testing, the test statistic (e.g. *F*, *t*, *r*) with confidence intervals, effect sizes, degrees of freedom and *P* value noted<br>*Give P values as exact values whenever suitable.*
☒	☐	For Bayesian analysis, information on the choice of priors and Markov chain Monte Carlo settings
☒	☐	For hierarchical and complex designs, identification of the appropriate level for tests and full reporting of outcomes
☐	☒	Estimates of effect sizes (e.g. Cohen's *d*, Pearson's *r*), indicating how they were calculated

Our web collection on statistics for biologists contains articles on many of the points above.

Software and code

Policy information about availability of computer code

Data collection	All data used in this study are preexisting data in standard formats; no special software was used to collect the data.
Data analysis	Anaconda with python 3.7; tensorflow2.0; custom codes are available at https://github.com/wangjuncongyu/covid19_recovery

For manuscripts utilizing custom algorithms or software that are central to the research but not yet described in published literature, software must be made available to editors and reviewers. We strongly encourage code deposition in a community repository (e.g. GitHub). See the Nature Portfolio guidelines for submitting code & software for further information.

Data

Policy information about availability of data

All manuscripts must include a data availability statement. This statement should provide the following information, where applicable:

- Accession codes, unique identifiers, or web links for publicly available datasets
- A description of any restrictions on data availability
- For clinical datasets or third party data, please ensure that the statement adheres to our policy

A total of 258 samples are published at https://github.com/wangjuncongyu/covid19_recovery; The whole survival dataset are not publicly available due to privacy concerns; However, researchers can contact the corresponding author to obtain the data upon reasonable request

March 2021

Field-specific reporting

Please select the one below that is the best fit for your research. If you are not sure, read the appropriate sections before making your selection.

☒ Life sciences ☐ Behavioural & social sciences ☐ Ecological, evolutionary & environmental sciences

For a reference copy of the document with all sections, see nature.com/documents/nr-reporting-summary-flat.pdf

Life sciences study design

All studies must disclose on these points even when the disclosure is negative.

Sample size	Data from a total of 3008 patients were collected from three hospitals (one for internal validation, the other two for external validation) in Wuhan, China, for large-scale verification. No formal sample size calculation was performed.
Data exclusions	The data exclusion criteria are described in detail in the Materials subsection. In general, patients with over 80% missed biomarkers were excluded. Besides, CT scans with slice thickness>3mm were excluded to reduce the heterogeneity.
Replication	We assessed reproducibility of the method through 5-fold cross validation using the interval dataset. Furthermore, external validation were performed to validate the generalization ability of the proposed method. All codes, pretrained models, and 258 samples were published for replication: https://github.com/wangjuncongyu/covid19_recovery
Randomization	The samples for internal validation were randomly divided into subsets for 5-fold cross validation
Blinding	No blinding test in this study

Reporting for specific materials, systems and methods

We require information from authors about some types of materials, experimental systems and methods used in many studies. Here, indicate whether each material, system or method listed is relevant to your study. If you are not sure if a list item applies to your research, read the appropriate section before selecting a response.

Materials & experimental systems

n/a	Involved in the study	
☒	☐	Antibodies
☒	☐	Eukaryotic cell lines
☒	☐	Palaeontology and archaeology
☒	☐	Animals and other organisms
☐	☒	Human research participants
☒	☐	Clinical data
☒	☐	Dual use research of concern

Methods

n/a	Involved in the study	
☒	☐	ChIP-seq
☒	☐	Flow cytometry
☒	☐	MRI-based neuroimaging

Human research participants

Policy information about studies involving human research participants

Population characteristics	This information is presented for each of the three patient cohorts in Supplementary Tables 1-3
Recruitment	Inclusion criteria for the three cohorts are presented in the Materials subsection. Confirmed cases of COVID-19 were defined as positive RT-PCR according to World Health Organization interim guidance. Strict recovery criteria were executed according to the diagnostic and treatment guideline for COVID-19 issued by the Chinese National Health Committee (version seventh).
Ethics oversight	The IRBs of the participating centers (Ethis Committee of the First Affiliated Hospital of Army Medical University, Approval Number: KY2020277) approved the study.

Note that full information on the approval of the study protocol must also be provided in the manuscript.
